# Low-Entropy Hydration Shells at the Spike RBD’s Binding Site May Reveal the Contagiousness of SARS-CoV-2 Variants

**DOI:** 10.3390/biom13111628

**Published:** 2023-11-07

**Authors:** Lin Yang, Shuai Guo, Chengyu Hou, Shenda Jiang, Liping Shi, Xiaoliang Ma, Bing Zheng, Yi Fang, Lin Ye, Xiaodong He

**Affiliations:** 1 National Key Laboratory of Science and Technology on Advanced Composites in Special Environments, Center for Composite Materials and Structures, Harbin Institute of Technology, Harbin 150080, China; 20b918032@stu.hit.edu.cn (S.G.); 22b918022@stu.hit.edu.cn (S.J.); shiliping@hit.edu.cn (L.S.); maxiaoliang@hit.edu.cn (X.M.); 2 School of Aerospace, Mechanical and Mechatronic Engineering, The University of Sydney, Sydney, NSW 2006, Australia; 3 School of Electronics and Information Engineering, Harbin Institute of Technology, Harbin 150080, China; houcy@hit.edu.cn; 4 Key Laboratory of Functional Inorganic Material Chemistry (Ministry of Education) and School of Chemistry and Materials Science, Heilongjiang University, Harbin 150001, China; zhengbing@hlju.edu.cn; 5 Department of Mathematics, Nanchang University, Nanchang 330031, China; yifang@ncu.edu.cn; 6 School of System Design and Intelligent Manufacturing, Southern University of Science and Technology, Shenzhen 518055, China; yelin@sustech.edu.cn; 7 Shenzhen STRONG Advanced Materials Research Institute Co., Ltd., Shenzhen 518035, China

**Keywords:** low-entropy hydration shell, protein–protein interaction, SARS-CoV-2 variants, contagiousness

## Abstract

The infectivity of the severe acute respiratory syndrome coronavirus 2 (SARS-CoV-2) is primarily determined by the binding affinity between the receptor-binding domain (RBD) of the spike protein and the angiotensin-converting enzyme 2 (ACE2) receptor. Here, through screening off pseudo hydrophilic groups on protein surfaces, the distribution of low-entropy regions on hydration shells of the ACE2 receptor and the RBDs of multiple SARS-CoV-2 variants was demonstrated. Shape matching between the low-entropy hydration shells of multiple SARS-CoV-2 variants and the ACE2 receptor has been identified as a mechanism that drives hydrophobic attraction between the RBDs and the ACE2 receptor, which estimates the binding affinity. Low-entropy regions of the hydration shells, which play important roles in determining the binding of other viruses and their receptors, are demonstrated. The RBD–ACE2 binding is thus found to be guided by hydrophobic collapse between the shape-matched low-entropy regions of the hydration shells of the proteins. A measure of the low-entropy status of the hydration shells can be estimated by calculating genuine hydrophilic groups within the binding sites. An important indicator of the contagiousness of SARS-CoV-2 variants is the low-entropy level of its hydration shells at the spike protein binding site.

## 1. Introduction

The severe acute respiratory syndrome coronavirus 2 (SARS-CoV-2) caused the COVID-19 pandemic and became a global concern. Previous studies have shown that the infectivity of SARS-CoV-2 mainly depends on the binding affinity of the receptor-binding domain (RBD) of the S protein to the angiotensin-converting enzyme 2 (ACE2) receptor [1,2,3]. It is worth noting that not all people were infected in a similar way. Several other factors, such as host cell proteases, polymorphisms of individual genes, and the glycosylation, interplay with the RBD–ACE2 binding affinity that influences sensitivity to COVID-19 [4,5,6,7]. Nonetheless, studying the interaction between RBD and ACE2 can help in understanding the infectivity of different virus variants. The contagious nature of SARS-CoV-2 arises from a specific physical binding between a spike protein of the coronavirus and a receptor protein on the host cell. Evidence obtained using cryo-electron microscopy (cryo-EM) showed that the backbone conformations of the RBD of the original SARS-CoV-2 S protein are almost the same as that of the SARS-CoV S protein [8]. Wrapp et al. revealed that the binding affinity of SARS-CoV-2 S RBD to ACE2 is more than 10-fold higher than SARS-CoV, which may contribute to the higher infectivity and transmissibility of SARS-CoV-2 compared to SARS-CoV [9]. Compared with SARS-CoV RBD, the SARS-CoV-2 RBD has more hydrophobic residue sidechains protruding outward at the interface with ACE2. The increment in hydrophobic interaction between RBD and ACE2 has been considered the reason for the infectivity of SARS-CoV-2 compared with SARS-CoV [10]. During the COVID-19 pandemic, the World Health Organization (WHO) classified several important SARS-CoV-2 variants as variants of interest (VOI) and variants of concern (VOC), and most amino acid mutations in VOIs and VOCs were located at the RBD binding site.

**Figure 1:**
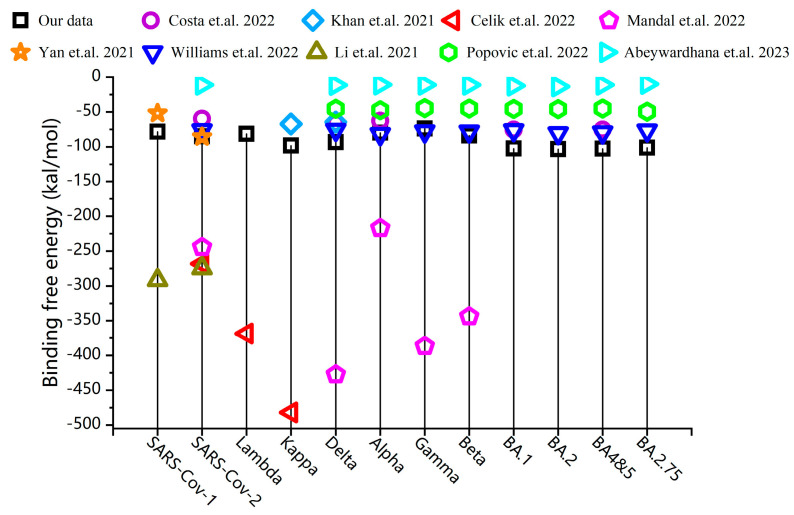
The RBD–ACE2 binding free energy for SARS-CoV-1 and SARS-CoV-2 variants, calculated by considering electrostatic interaction, van der Waals interaction, polar solvation energy, and non-polar solvation energy, compared with the binding energies reported in other references [12,13,14,15,16,17,18,19,20].

Despite significant theoretical and experimental efforts by many scientists, the physical mechanism of protein–protein binding still remains unclear. To determine the binding free energy between RBD and ACE2, the molecular mechanics Poisson–Boltzmann surface area (MM–PBSA) computational method is usually employed to dissect the effect and affinity of SARS-CoV-2 variants RBD and ACE2. In this article, the RBD–ACE2 binding free energies of major SARS-CoV-2 variants were calculated using the MM–PBSA, which took into consideration electrostatic, van der Waals, hydrogen bond interactions, polar solvation energy, and non-polar solvation energy (see Figure 1). The binding free energy of the RBD–ACE2 complexes calculated using Gromacs 2021.1 software is illustrated in Figure 1 (see the Supplementary details) [11]. Given the significant differences in results reported in different studies that have been referred to here as shown in Figure 1, we found that the binding free energy of RBD–ACE2 does not accurately describe the differences in contagious efficiency among the SARS-CoV-2 variants [12,13,14,15,16,17,18,19,20].

As a typical spontaneous reaction, protein–protein binding has been considered to be driven by hydrogen bonding, electrostatic, van der Waals, and hydrophobic interaction forces [21,22]. However, our new study suggests that electrostatic interaction and hydrogen bonding between the two proteins do not dominate the approaching stage of protein–protein binding, as the electrostatic interactions between two proteins are most likely to be blocked by the polar water molecules [23]. Consequently, water-mediated hydrophobic interactions are a pivotal regulatory mechanism in the protein–protein binding process, and their role is far more important than hydrogen bonding, electrostatic, and van der Waals interactions.

The hydration shells around the protein surface exhibited different dynamics and structures from bulk water at a distance of approximately 1–5 nm [10,23]. Therefore, the hydrophobic interactions between two proteins may begin to take effect within a distance range of a few nanometers. Simulation and experimental studies have also shown that the speed of water molecules is greatly reduced by 2–10 times when they enter the hydration shell of a protein [24,25]. The reported evidence has shown that the protein hydration shells have a more ordered hydrogen-bonding network than bulk water molecules [26,27], and therefore, the low-entropy level of the hydration shell is lower than that of bulk water [28,29]. Recent research has reported that the dynamics of a hydration shell are highly heterogeneous and that hydrophobic regions favor a more tetrahedral water structure and less entropy [27,30].

Moreover, the prerequisite for electrostatic, hydrogen bond, and van der Waals interactions between two proteins is that hydrophilic groups of protein surface at the binding sites do not form hydrogen bonds with the surrounding polar water molecules [31,32,33,34]. Therefore, water-mediated hydrophobic interactions, which are long-range intermolecular attractions, are dominant in the process of protein recognition and proximity at longer distances. As a consequence, protein–protein binding should be mainly guided by the water-mediated hydrophobic interactions between the low-entropy regions of the hydration shells of individual proteins. However, practical methods for identifying low-entropy hydration shells of proteins have been rarely reported. In our past work, we proposed a new protein–protein docking mechanism based on an innovative approach to identifying low-entropy hydration shells of proteins. In the present work, we provide potential novel biophysical insights into the low-entropy hydration shells of protein–protein binding sites and the link between the contagious nature of SARS-CoV-2 variants and the low-entropy level of its hydration shells at the spike RBD’s binding site.

## 2. Materials and Methods

### 2.1. Protein Structures

In the present work, the experimentally determined native structures of proteins were sourced from the Protein Data Bank (PDB), which is a database for the three-dimensional structural information of large biological molecules, such as proteins [35]. We used 334 experimental protein structures containing 200 SARS-CoV-2 RBD antibody structures and 10 variants of concern to dissect the contagiousness of SARS-CoV-2 variants. The PDBIDs for all proteins are listed in all figures. We identified the morphology of low-entropy hydration shells on the protein surface at the binding site by the visualization software PyMOL (version 2.5) [36]. Mutations in the RBDs of the spikes of SARS-CoV-2 variants are illustrated in Supplementary Figure S1.

### 2.2. Hydrophilicity of Residues

The detailed hydrophilicity of 20 residues can be easily identified from the partial charges based on the topological structures in charmm36 force field [37]. The partial charge of the residues can be used as an indicator of the hydrophilicity for each atom (see Supplementary Figure S2). For example, the alkyl group is highly hydrophobic, making it difficult to form hydrogen bonds with water molecules. Their partial charges, which can help locate hydrophobic areas on the protein, mainly range from −0.27 e to −0.09 e, according to which the carbon atom is considered hydrophobic. In contrast, due to the high electronegativity of nitrogen atoms (mainly from −0.47 e to −0.8 e) and oxygen atoms (from −0.51 e to −0.76 e), the oxygen and nitrogen atoms that form hydrogen bonds with surrounding water molecules are considered hydrophilic.

### 2.3. Defining the Low-Entropy Level of the Hydration Shells

The low-entropy hydration shells of protein surface at the binding site are not simply determined by the distribution of hydrophilic and hydrophobic groups. If the hydrophilic groups do not express their hydrophilicity, that is, the hydration shell of the hydrophilic groups is low-entropy, they facilitate the formation of low-entropy hydration shells around protein surfaces. We identify the low-entropy hydration shell regions on the protein surfaces via three steps.

A custom Python script has been compiled to directly resolve experimental protein complexes and automatically identify the low-entropy hydration shell regions. First, the hydrogen bonds formed by the hydrophilic groups on the protein surface are saturated, thereby preventing these hydrophilic groups from forming hydrogen bonds with surrounding water molecules [38]. When an intramolecular hydrogen-bonded hydrophilic group is located on the protein surface, we thus consider that the hydration shell of that hydrophilic group is a low-entropy hydration shell.

Second, when the sidechains of leucine, isoleucine, valine, tryptophan, phenylalanine, tyrosine, alanine, methionine, cysteine, and proline residues are located at the binding site and protrude outward to the surrounding aqueous solution, they can prevent the backbone carbonyl oxygen atoms and amide hydrogen atoms of the residue from interacting with the water molecules. The sidechains of lysine and arginine residues contain hydrophobic structures (i.e., alkyl) between the hydrophilic tops of the sidechains and the backbones that can also reduce and hinder the exchange of hydrogen bonds between backbone atoms and water molecules. The hydration shells surrounding the backbone carbonyl oxygen groups and the amide hydrogen groups of these residues should also be considered low-entropy hydration shells, i.e., pseudo hydrophilicity. Thus, the hydration shells of backbone carbonyl oxygen atoms and the amide hydrogen atoms of these residues were considered low-entropy hydration shells.

Third, according to the hydrophobicity scales of amino acid residues, tryptophan and tyrosine are classified as hydrophobic residues with long sidechains [39,40,41]. This is because the long sidechains of tryptophan and tyrosine residues are mainly composed of hydrophobic alkyl and benzene ring structures. Neither of the two residues can substantially express hydrophilicity through the small CO or NH groups at the end of the hydrophobic sidechains because most neighboring water molecules have been fixed in the long hydrophobic residue, inducing an ordered network [38]. Lysine has a long sidechain composed of the non-polar hydrophobic and polar hydrophilic part. The hydrophobic sidechain can reduce and hinder the exchange of hydrogen bonds between a few hydrophilic atoms near it and water molecules. Therefore, the hydration shells surrounding tryptophan, tyrosine, and lysine can be considered low-entropy hydration shells.

Therefore, after excluding the hydrophilic groups that do not express hydrophilicity, only the remaining hydrophilic groups can express hydrophilicity. The proportion of the surface area occupied by hydrophilic groups in the low-entropy regions of the hydration shells at the binding site can serve as an indicator of the contagiousness of the coronavirus and its variants.

## 3. Results

### 3.1. Low-Entropy Regions of Hydration Shells of Proteins

**Figure 2:**
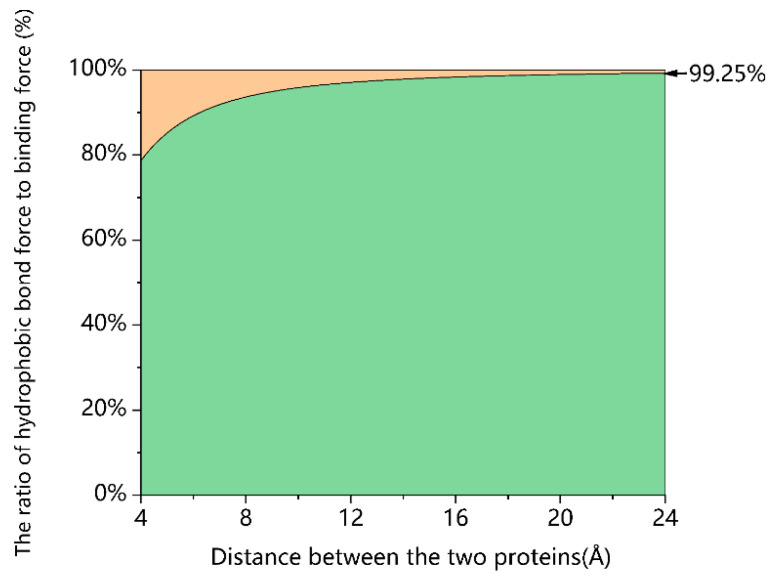
Percentage share of hydrophobic interaction force in the overall attractive force between the ACE2 receptor and the SARS-CoV-2 RBD during the binding process.

Several studies have shown that the standard molar entropy of ordered water structures around non-polar surfaces is approximately equal to the standard molar entropy (41 J/mol/K) of solid water, and that of liquid water is approximately 70 J/mol/K [42]. Thus, the movement of an ordered water molecule from a low-entropy hydration shell to disordered liquid can produce a molar entropy difference (∆S) of 29 J/mol/K. For the hydration shell with one ordered water molecule removed, the entropy increase (TΔS) is 8961 J/mol at 309 K. The density of the protein hydration shells with ordered water molecules is approximately 33 water molecules per 1 nm^3^. The hydrophobic surface area of SARS-CoV-2 RBD involved in binding with the ACE2 receptor is approximately 867.4 Å^2^ [10]; the hydrophobic attractive forces between the ACE2 receptor and RBD are approximately 4.19 nN at a starting distance of approximately 2 nm. The sum of the electrostatic, van der Waals, and hydrogen bond interactions between the ACE2 receptor and RBD at a distance can be calculated using the following equation:

(1) $$F=F_{e}+F_{vw}+F_{hb}\approx \sum \frac{KQ_{j}Q_{k}}{r^{2}}+\sum \nabla_{r}(-\frac{A}{r^{6}}+\frac{B}{r^{12}})+\sum \nabla_{r}\{[\frac{C}{r^{6}}-\frac{D}{r^{4}}]{cos}^{4}\theta \}$$

where *F_e_* is the electrostatic forces, *F_vw_* is the van der Waals forces, *F_hb_* is the hydrogen bonds, *Q_j_*, *Q_k_* is the amount of charge, *K* is the electrostatic force constant, and *θ* is the hydrogen donor–acceptor angle. *A*, *B*, *C*, and *D* are the parameters determined by the atom, and *r* is the distance between the charged atoms in the proteins. The sum of the hydrogen bonding, electrostatic attraction, and van der Waals forces between the ACE2 receptor and RBD at a distance of 2 nm is approximately 0.032 nN, which is negligible compared to the hydrophobic interaction force. When the distance between the ACE2 receptor and RBD ranges from 0.7 nm to 2.4 nm, the hydrophobic interaction force accounts for more than 90% of the attractive force between the ACE2 receptor and RBD (see Figure 2).

The dynamics of the hydration shell on the protein surface are not simply determined by the spatial distribution of hydrophilic and hydrophobic residues [43,44]. As a typical spontaneous reaction, hydration shells around binding sites must gradually be squeezed out at the binding sites in the approaching stage of protein–protein binding; increased entropy of the water molecules is inevitable. Therefore, binding sites on proteins should be generally covered by large low-entropy regions of the hydration shell to trigger long-range intermolecular hydrophobic attractions between proteins. The distribution of hydrophilic and hydrophobic groups on the binding sites of proteins appears disorderly; the hydrophilicity of a hydrophilic group is expressed through hydrogen bonding with surrounding water molecules. If a hydrophilic group cannot form hydrogen bonds with the surrounding water molecules, we consider that the hydrophilic group cannot express its hydrophilicity and that these intramolecular hydrogen-bonded hydrophilic groups are pseudo hydrophilic.

The reasons why the hydrophilic groups on the protein surface do not express hydrophilicity are summarized in the following. First, the hydrogen bonds formed by the hydrophilic groups on the protein surface are saturated, thereby preventing these hydrophilic groups from hydrogen bonding with the surrounding water molecules [38]. Thus, when an intramolecular hydrogen-bonded hydrophilic group is located on the protein surface, the hydrophilicity of the hydrophilic group is negligible. Second, when a hydrophobic sidechain is located at the binding site of the protein, it can prevent the hydrophilicity of the backbone atoms of these residues from the surrounding water molecules. Third, based on the hydrophobicity scales of amino acid residues, tryptophan and tyrosine are classified as hydrophobic residues with long sidechains [39,40,45]. The hydrophilicity of the O and N atoms in the long sidechains of tryptophan, tyrosine, and lysine should also be statistically undetectable. This is because the long sidechains of tryptophan and tyrosine residues are mainly composed of hydrophobic alkyl and benzene ring structures. Therefore, the hydration shells surrounding tryptophan, tyrosine, and lysine can be considered low-entropy hydration shells.

**Figure 3:**
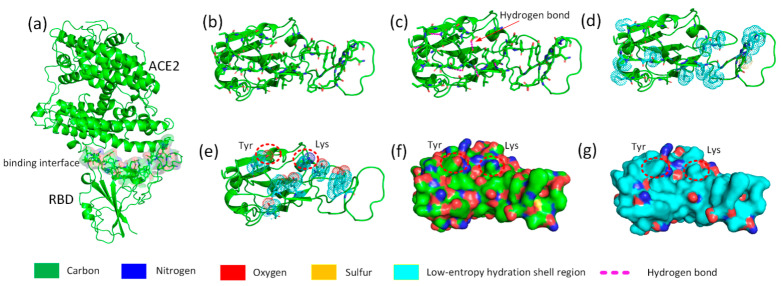
Schematic workflow of screening off pseudo hydrophilic groups on the binding site of the SARS-CoV-2 S RBD. (**a**) Molecular structure of SARS-CoV-2 RBD and ACE2; the following figures exhibit only the RBD binding interface. (**b**) Amino acids adjacent to ACE2 on the RBD binding interface. (**c**) Intramolecular hydrogen bonding on the RBD binding interface (highlighted by red dashed lines). (**d**) Hydrophobic sidechains protruding on the surface of the protein (carbon atoms are highlighted by cyan dots). (**e**) The distribution of tyrosine and lysine on the RBD binding interface (oxygen, nitrogen, and carbon atoms are highlighted by red, blue, and cyan dots, respectively). (**f**) The topography distribution of hydrophilic nitrogen and oxygen atoms on the normal RBD surface. (**g**) The topography distribution of the low-entropy hydration shell on RBD surface (highlighted in cyan).

Using the above method, we screened and eliminated the pseudo hydrophilic groups on the binding site of the SARS-CoV-2 S RBD. The three steps of this method are illustrated in Figure 3c–e. The surfaces of the RBD before and after the screening off of pseudo hydrophilic groups are illustrated in Figure 3f and Figure 3g, respectively. This innovative method reveals that large low-entropy regions of the hydration shells generally occupy the binding sites of proteins.

### 3.2. The Low-Entropy Hydration Shells of SARS-CoV-2 RBD and ACE2

In this section, we further analyze the role that the low-entropy hydration shells played in protein–protein binding. Protein–protein binding should be dominated by the long-range hydrophobic attraction which is generated by the low-entropy regions of the hydration shells of the proteins [46,47]. Although the distribution of hydrophilic and hydrophobic groups on the binding sites of proteins appears disorderly, the hydration shell regions that cover the binding sites of proteins may be described as having low entropy.

**Figure 4:**
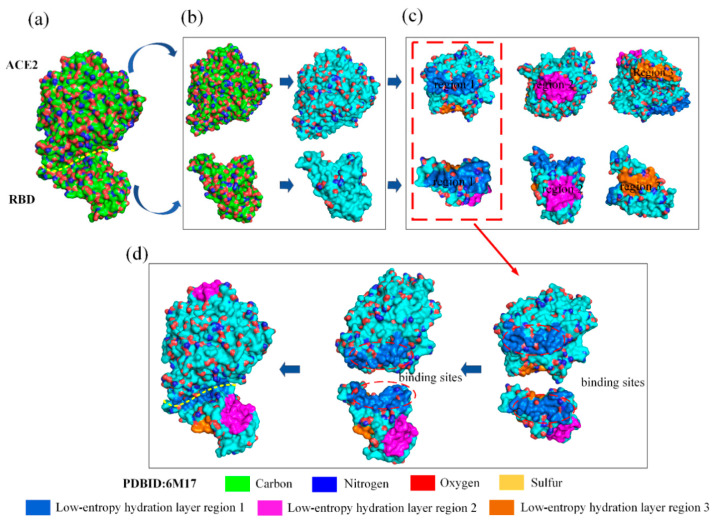
Low-entropy hydration shells on the binding sites of SARS-CoV-2 RBD and ACE2. (**a**) The normal surface of ACE2 and SARS-CoV-2 RBD. (**b**) The low-entropy hydration shell regions of RBD and ACE2 (highlighted in cyan). (**c**) Three larger low-entropy hydration shell regions of RBD and ACE2 are depicted in marine (region 1), magenta (region 2), and orange (region 3). (**d**) The larger low-entropy regions of hydration shells covering the binding sites of RBD and ACE2.

The low-entropy regions of the hydration shell of the RBD–ACE2 complex can be mapped by searching for pseudo hydrophilic and hydrophobic groups on the protein surface via the three steps described above (see Figure 4). The detailed operation procedure of mapping the low-entropy regions of the hydration shells occupying the binding sites of the RBD and ACE2 is illustrated in Supplementary Figure S3. Several large low-entropy regions of the hydration shells on the entire surface of RBD and ACE2 were observed, and only two such regions at the binding site had perfectly matching shapes based on the results of analysis using fast Fourier transform (FFT) methods. It can be concluded that low-entropy hydration shells induced by hydrophobic groups can cover neighboring intramolecular hydrogen-bonded hydrophilic groups on the protein surface, owing to the hydrophilicity of these hydrophilic groups being expressed by the intramolecular hydrogen bonds. The result indicates that RBD–ACE2 binding is guided by the hydrophobic collapse between the shape-matched low-entropy regions of the hydration shells.

### 3.3. Verification with Test System

**Figure 5:**
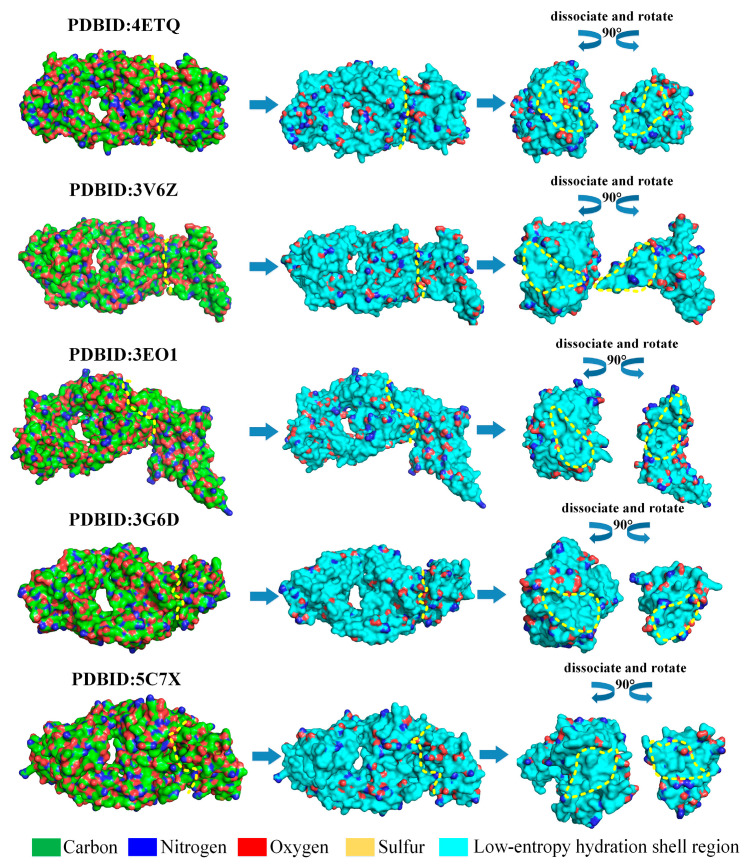
Low-entropy hydration shells at binding sites guide protein binding using five antibody–antigen test cases for Docking Benchmark 5.5. The low-entropy hydration shell regions are shown in cyan, and the curves and irregular circles marked with yellow dashed lines represent the binding surface and binding sites, respectively.

Binding sites on proteins should be covered by large low-entropy regions of the hydration shell. To prove this, a Python script program has been compiled to resolve experimental protein complexes and screen off pseudo hydrophilic groups to map the low-entropy hydration shell regions on the protein surfaces. In the present work, to illustrate low-entropy hydration shells at binding sites as a universal mechanism guiding protein binding, the low-entropy hydration shell regions of all the 67 antibody–antigen test cases for Docking Benchmark 5.5 released in 2021 are mapped by eliminating pseudo hydrophilic groups, as illustrated in Figure 5 and Figure S4 [48]. It is obvious that hydrophilic groups located at the binding sites of proteins normally do not express their hydrophilicity in order to ensure that protein binding sites are generally occupied by large low-entropy regions of hydration shells. The 67 antibody–antigen testing cases illustrate that low-entropy hydration shells covering the binding site is a universal phenomenon.

To further verify the low-entropy hydration shell theory, we mapped the low-entropy regions of the hydration shells for another 50 protein quaternary structures, and found that shape matching between the low-entropy hydration shells of protein binding sites is a widespread phenomenon (see Supplementary Figure S5). All 50 protein quaternary structures were randomly selected from the Protein Data Bank (PDB) [35]. The low-entropy hydration shell inducing protein docking is prevalent in all investigated protein quaternary structures. This indicates that protein–protein binding is not random but mainly dominated by entropy increase between the shape-matched low-entropy regions of hydration shells of individual proteins [10,49]. The spatial layout of the low-entropy hydration shells of the protein is similar to a ‘lock and key’ mechanism to guide the protein–protein binding in a precise manner. It is worth noting that protein–protein binding affinity may be assessed by the low-entropy level of the hydration shell at the binding sites.

### 3.4. The Low-Entropy States of Hydration Shell at the Binding Sites

**Figure 6:**
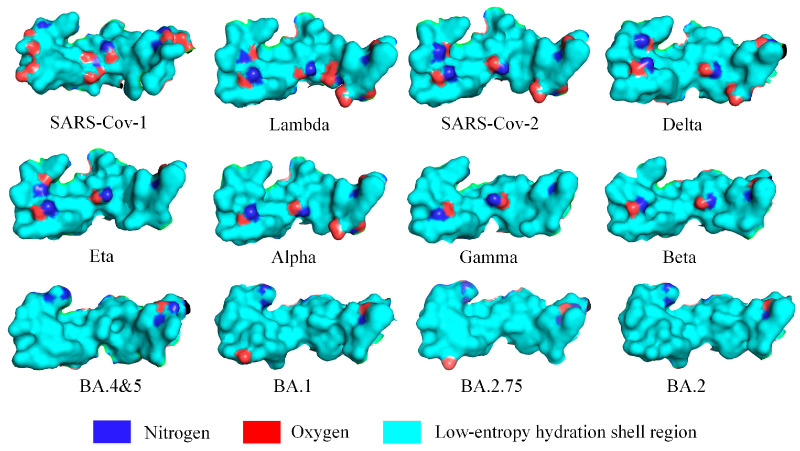
Distribution of the surface area of expressed hydrophilic groups on the low-entropy hydration shells at the RBD binding sites of SARS-CoV-1, SARS-CoV-2, and SARS-CoV-2 variants with the ACE2 receptor. The low-entropy region of the hydration shells is shown in cyan. PDBIDs include: 2AJF, 7FEM, 6M17, 7W9I, 7NXC, 7R11, 7WBL, 7XWA, 8ASY, etc.

Since the COVID-19 outbreak, SARS-CoV-2 has been continuously evolving and generating new variants, meaning that the low-entropy regions of hydration shells at the RBD binding site are undergoing important changes. Therefore, changes in the low-entropy status of the hydration shells of the spike RBD’s binding site most likely remarkably influence the level of contagiousness of SARS-CoV-2 variants. As observed at the wild–type SARS-CoV-2 RBD binding site, there are multiple isolated hydrophilic groups that still exhibit hydrophilicity. A hydrophilic group within a low-entropy hydration shell region inevitably exchanges its hydrogen bonding with different surrounding water molecules, disrupting the low-entropy state of the low-entropy hydration shell. Therefore, we use the proportion of the surface area of expressed hydrophilic groups in the low-entropy region at the binding sites as an indicator to assess the binding affinity of two protein partners. Therefore, a lower level of entropy of the hydration shell at the binding site is most likely beneficial to the binding affinity and consequently facilitates the transmission of the SARS-CoV-2 variant. We drew the low-entropy hydration shell regions at the RBD binding sites of SARS-CoV, SARS-CoV-2, and 10 SARS-CoV-2 variants in detail (see Figure 6). The RBD of the Omicron BA.2 variant is derived from a mutation to the RBD of the Omicron BA.1. It is worth noting that glycosylation sites on the RDB of the spike protein are not located at the binding site with ACE2. Normally, glycosylation sites should not be included within the low-entropy hydration shell regions unless the glycosylation site is surrounded by highly hydrophobic sidechains at the surface.

**Figure 7:**
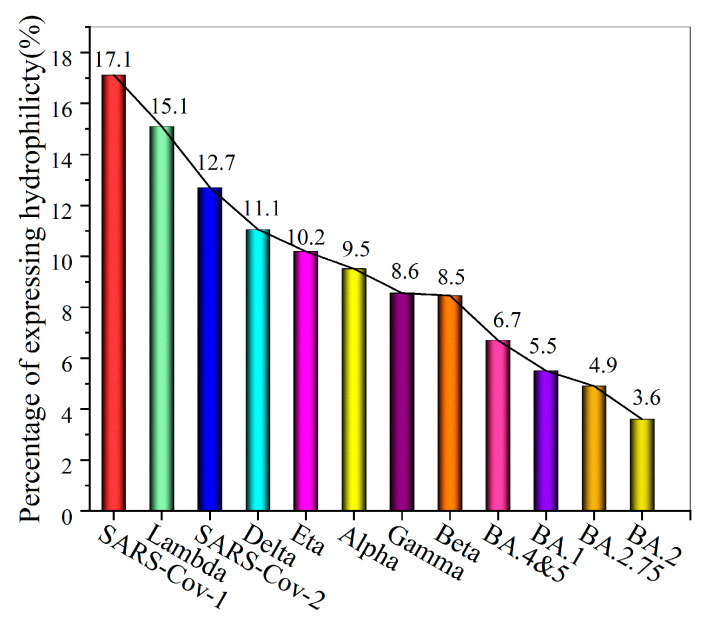
The proportion of the surface area of expressed hydrophilic groups on the low-entropy hydration shells at the RBD binding sites of SARS-CoV-1, SARS-CoV-2, and 10 SARS-CoV-2 variants.

The proportions of the surface areas of the expressed hydrophilic groups on the low-entropy hydration shells at the RBD binding sites are illustrated in Figure 7. A lower proportion indicates a lower entropy level for the hydration shell at the binding sites. We found that the more recent the coronavirus variant, the stronger the capability to infect and transmit among humans, and the fewer the hydrophilic groups that can express their hydrophilicity at the binding site of the RBD. Therefore, a lower level of entropy of the hydration shell at the binding site most likely leads to a higher binding affinity and consequently facilitates the transmission of the SARS-CoV-2 variant. The proportions of the surface area of the expressed hydrophilic groups on the low-entropy hydration shells at the RBD binding sites showed that the contagiousness of these variants descended in the given order: SARS-CoV-1, Lambda, SARS-CoV-2, Delta, Eta, Alpha, Gamma, BA.4 and 5, Omicron BA.1, BA.2.75, and BA.2. Interestingly, these results are in agreement with the differences in the contagiousness of the different variants deduced from epidemiological statistics [31,50,51,52,53,54,55,56,57].

**Figure 8:**
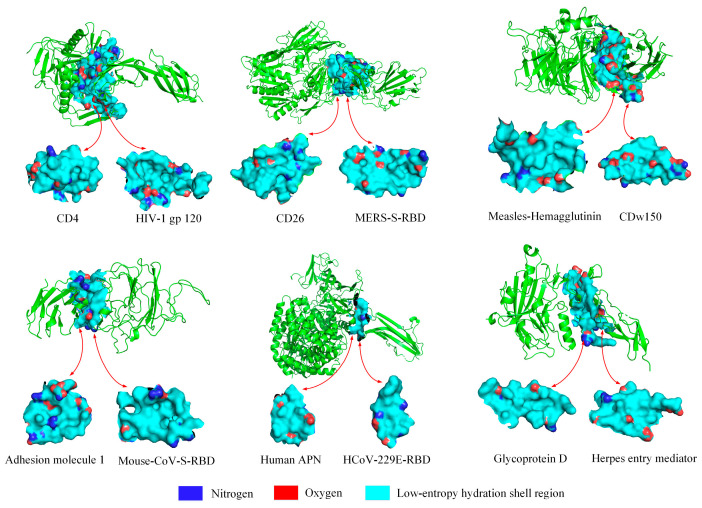
Expressed hydrophilic groups in the low-entropy regions of hydration shells at the binding sites of six other viruses. The six viruses are HIV (PDBID: 4RQS), MERS (PDBID: 4KR0), mouse coronavirus (PDBID: 6VSJ), measles virus hemagglutinin (PDBID: 3ALZ), human coronavirus HCoV-229E (PDBID: 6U7G), and herpes (PDBID: 1JMA), respectively.

**Figure 9:**
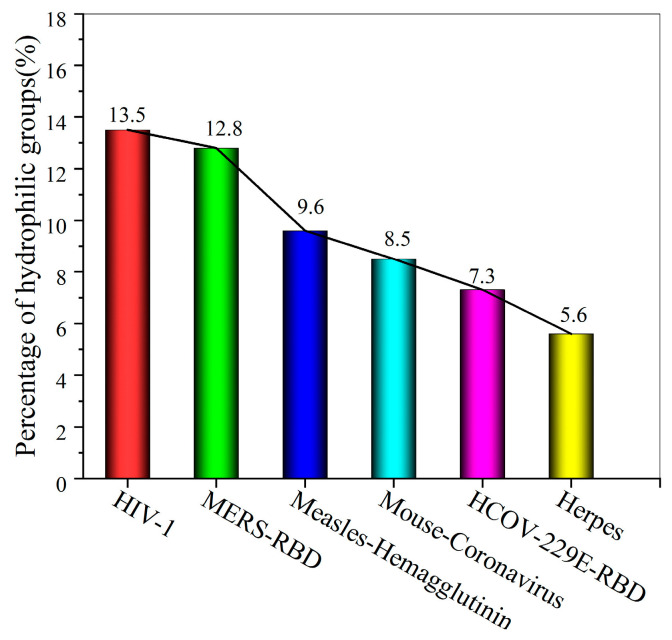
Percentage surface area occupied by hydrophilic groups expressed in the low-entropy region at the binding sites of six viruses.

To further show that the low-entropy level of the hydration shell at the binding site is an important index reflecting the transmission efficiency of different variants, the proportions of the surface areas of the expressed hydrophilic groups at the binding sites of six other viruses were analyzed, as shown in Figure 8 and Figure 9. The low-entropy hydration shells at the binding sites of six other viruses are depicted in Supplementary Figure S6. The proportion of the surface area of the expressed hydrophilic groups on the low-entropy hydration shell regions may further help us understand the contagiousness of viruses.

**Figure 10:**
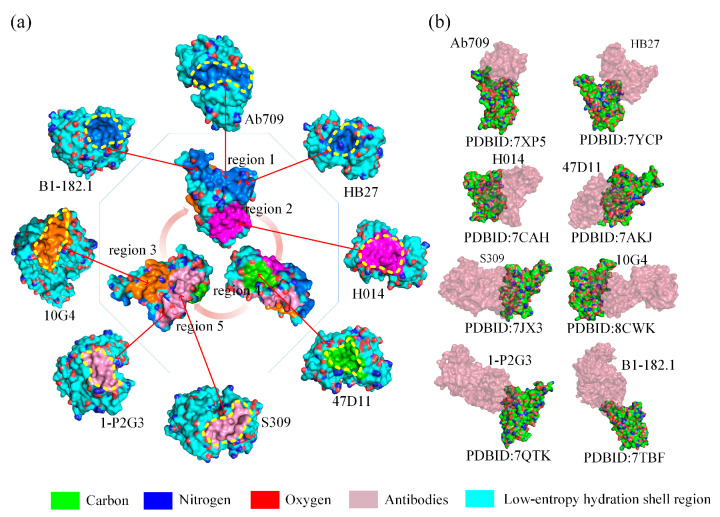
(**a**) A schematic plot of the eight representative antibodies bound to five low-entropy regions of hydration shells on the surface of the SARS-CoV-2 spike protein RBD; The contour of the binding site is a yellow dashed circle.: marine (region 1), magenta (region 2), orange (region 3), chartreuse (region 4), and pink (region 5). (**b**) Schematic representation of eight representative antibodies bound to SARS-CoV-2 spike protein RBD; the antibodies are shown in transparent burgundy.

According to the World Health Organization COVID-19 dashboard, SARS-CoV-2 has been responsible for more than 6.9 million deaths to date. The vaccine–elicited antibodies are powerful tools to tackle the pandemic. For the design of effective neutralizing antibodies, the first consideration is to identify the target sites, and then consider how to enhance the binding affinity. By screening off pseudo hydrophilic groups, we mapped five low-entropy hydration shells on the surface of the SARS-CoV-2 spike protein RBD. We studied 200 experimentally determined native structures of antibodies specific to RBD in the PDB database using the low-entropy hydration shells theory. The results show that all the different antibodies bind to any one of the five low-entropy regions of the hydration shells as shown in Figure 10a. Visual structural conformations of eight antibodies bound to the RBD sites are shown schematically in Figure 10b. All the statistical data of RBD–antibody binding sites are listed in Supplementary Table S1. We found that 128 antibodies bind at region 1, 49 antibodies bind at region 2, 24 antibodies bind at region 3, 5 antibodies bind at region 4, and 47 antibodies bind at region 5, respectively. The statistical results proved that the binding sites of these antibodies are indeed not randomly distributed, indicating that protein–protein binding is predominantly driven by hydrophobic attraction at the binding sites, which eventually leads to shape matching of their low-entropy hydration shells at the binding sites.

**Figure 11:**
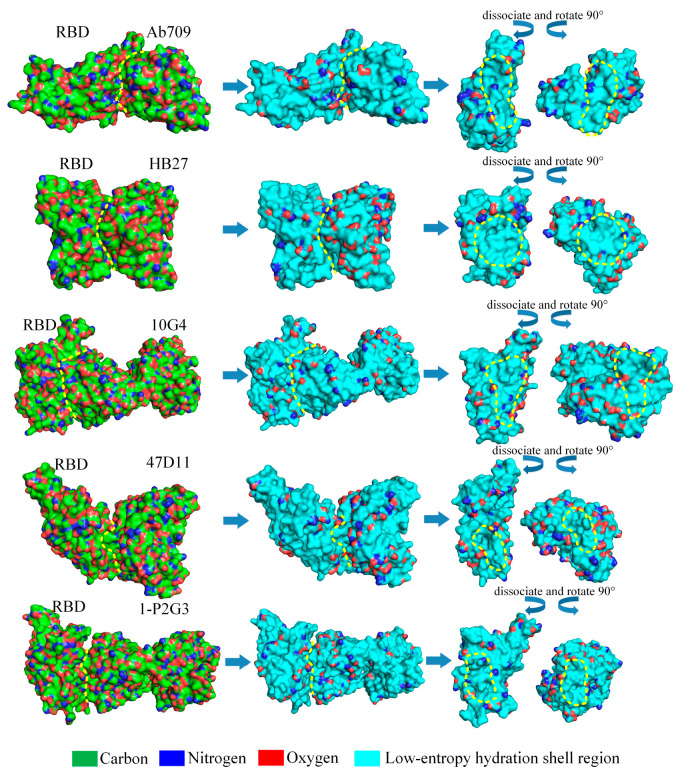
Schematic illustration of the proportions of the expressed hydrophilic groups in the low-entropy region of Ab709, H104, 10G4, and S309 antibodies and RBD at binding sites. The low-entropy hydration shell regions are shown in cyan; the curves and irregular circles marked with yellow dashed lines represent the binding surface and binding sites, respectively.

The low-entropy hydration shells theory is important for the provision of reliable therapeutic target sites for neutralizing antibodies and assessment of the binding affinity, which accelerates antibody drug research and design because the proportions of the surface area of the expressed hydrophilic groups in the low-entropy region at the binding sites may impact the contagiousness of these variants. The Ab709, H104, 10G4, and S309 antibodies present a typical low-entropy status of the hydration shell at the binding sites without expressing hydrophilic groups; therefore, these antibodies should be excellent options for halting the spread of COVID-19 (see Figure 11).

## 4. Conclusions

Not all hydrophilic groups at the binding site of a protein will express hydrophilicity, which can promote the formation of low-entropy hydration shell regions at the binding sites. Basically, protein–protein binding is coordinated in the rotational–conformational space of mutual orientations by the low-entropy regions of hydration shells on the protein surfaces, which may help to assess their binding affinity. The driving force of SARS-CoV 2 RBD and the ACE2 receptor is caused by long-range hydrophobic interaction between the two low-entropy hydration shells at the two binding sites. The entropy increase caused by the hydrophobic collapse of the low-entropy hydration shells provides the force for protein docking. In our past research, the low-entropy hydration shell theory answered the question, “How do proteins find their partners?” In the present study, this theory may reveal a new perspective of explanation and exploration regarding the contagiousness of SARS-CoV-2 variants. The low entropy of the hydration shell at the binding site can be evaluated by calculating the proportion of surface area of the genuine hydrophilic groups expressed at the binding site. The low-entropy level of the hydration shell at the binding site may be an important indicator to reveal the contagiousness of a coronavirus. The method to measure the low-entropy status of the hydration shells of proteins can enhance the understanding of pathogenesis, evaluation of virus infection, and antibody design.

## 5. Patents

The authors declare that the methods of low-entropy hydration shell used in this paper are protected by our patents, the Chinese Patent (No: CN202210138581.X.), American patent (No: US17/984,660), and European patent (No: EP23151692.3) “Method and device for protein-protein docking based on identification of low–entropy hydration layer on protein surface”.

## Data Availability

All relevant data are within the paper and Supplementary Materials.

## Supplementary Materials

The following supporting information can be downloaded at: https://www.mdpi.com/article/10.3390/biom13111628/s1, Figure S1: The mutations of SARS-CoV-1 and 11 different SARS-CoV-2 variants are of interest to scientists; Figure S2: The partial charges based on the topological structures in charmm36 force field; Figure S3: Low-entropy hydration of hydration shells on the binding sites of SARS-CoV-2 RBD and ACE2; Figure S4: Low–entropy hydration shells at binding sites guide protein binding using 62 antibody–antigen test cases for Docking Benchmark 5.5; Figure S5: The prediction of binding sites of protein subunits for 50 protein quaternary structures through identifying the low-entropy regions of hydration shells of individual protein subunits; Figure S6: The low-entropy region at the binding sites of HIV (PDBID: 4RQS), MERS (PDBID: 4KR0), mouse coronavirus (PDBID: 6VSJ), measles virus hemagglutinin (PDBID: 3ALZ), human coronavirus HCoV-229E (PDBID: 6U7G), and herpes (PDBID: 1JMA); Table S1: The statistical results of RBD-antibodies binding sites at 200 experimentally determined structures [8,11,36,58,59,60,61,62,63,64,65,66,67,68].
