# Supplementary material for: Low-Entropy Hydration Shells at the Spike RBD’s Binding Site May Reveal the Contagiousness of SARS-CoV-2 Variants
Source: Biomolecules. 2023 Nov 7;13(11):1628. doi: 10.3390/biom13111628 (PMC10669249; doi:10.3390/biom13111628)
Supplement: Supplementary file 1 [file biomolecules-13-01628-s001.zip › biomolecules-2583614-supplementary.pdf]

Supplementary

Molecular dynamics simulations of the binding free energy

To assess the binding free energy of the SARS-CoV Spike-RBD/ACE2 complex, we performed all-atom MD simulations of Wild-type (WT) and variants. Protein complexes were considered in this work, namely SARS-CoV-1 Spike-RBD/ACE2 (PDB ID: 2AJF) [1], SARS-CoV-2 Spike-RBD/ACE2 (PDB ID: 6M0J) [2], Alpha (B.1.1.7)、Beta (B.1.351) Spike-RBD/ACE2 (PDB ID: 7R10、7R11) [3], Delta (B.1.617.2) Spike-RBD/ACE2 (PDB ID: 7W9I) [4], Gamma (P.1) Spike-RBD/ACE2 (PDB ID: 7NXC) [5], Kappa Variant Spike-RBD/ACE2 (PDB ID: 7VX5) [6], Omicron BA.1 Spike-RBD/ACE2 (PDB ID: 7R10) [7]. The variants namely, Eta (B.1.525), Lambda (C.37), Mu (B.1.621) and Omicron) (BA.2) were generated by mutating specific residues in Wild-type Spike RBD using Pymol [8].

All atomistic MD simulation was carried out using Gromacs 2021.1 software[9] using Amber ff14SB force field parameters [10] and TIP3P water model [11]. A cubic simulation box consisting of 0.15mM NaCl was created for the systems under periodic boundary conditions (PBCs). The distance from the protein solute complex to the wall of the cube was set to 10 Å. The cutoff values of short-range nonbonded interactions were set to 12 Å. Particle-mesh Ewald method [12] was used to calculate the long-range electrostatic interactions. The systems were equilibrated at 300 K in NVT ensemble using V-rescale coupling algorithm for about 500ps and then equilibrated in NPT ensemble using Parrinello-Rahman barostat for 500ps [13]. Production simulation was carried out for the 12 protein complexes for an integration timestep of 2 fs and 50 ns in NPT.

The binding free energy between RBD and ACE2 for WT and variants were computed by using the Molecular Mechanics/Poisson Boltzmann Surface Area (MM/PBSA) employed in the gmx_MMPBSA tool [14], which is an end-point method. In this methodology, the binding free energy (ΔGbind) between the proteins is calculated by

$$\Delta G_{\mathrm{bind}} = \Delta G_{\mathrm{complex}} - \Delta G_{\mathrm{recepter}} - \Delta G_{\mathrm{ligand}}$$

$$\Delta G_{\mathrm{bind}} = \Delta H - T\Delta S \approx \Delta E_{\mathrm{MM}} + \Delta G_{\mathrm{solv}} - T\Delta S$$

$$\Delta E_{\mathrm{MM}} = \Delta E_{\mathrm{bond}} + \Delta E_{\mathrm{angle}} + \Delta E_{\mathrm{dihedral}} + \Delta E_{\mathrm{vdw}} + \Delta E_{\mathrm{coulomb}}$$

$$\Delta G_{\mathrm{solv}} = \Delta G_{\mathrm{polar}} + \Delta G_{\mathrm{nonpolar}}$$

where $\Delta G_{\mathrm{complex}}$, $\Delta G_{\mathrm{recepter}}$, and $\Delta G_{\mathrm{ligand}}$ represent the total free energies of the complex, the receptor, and the ligand, respectively. Further, ΔGbind can be usually decomposed into three terms: the average molecular mechanic's potential energy in the vacuum ΔE_{MM}, the free energy of solvation ΔGsol, and the conformational entropy $-T\ \Delta S$, here S and T denote the entropy and temperature, respectively. The ΔE_{MM} consists of bonded terms which include the bond stretching ΔE_{bond}, angle bending $\Delta E_{\mathrm{angle}}$ and dihedral angles $\Delta E_{\mathrm{dihedral}}$, and nonbonded terms, which include the electrostatic $\Delta E_{\mathrm{coulomb}}$ and the Van der waal interactions ΔE_{vdw}. The solvation free energy, ΔG_{solv} takes both electrostatic and non-electrostatic ($\Delta G_{\mathrm{polar}}$ and $\Delta G_{\mathrm{nonpolar}}$) components. The vacuum electrostatic dielectric constant and the solvent dielectric constant were set to 2 and 80, respectively.

1. Li, F.; Li, W.; Farzan, M.; Harrison, S.C. Structure of SARS coronavirus spike receptor-binding domain complexed with receptor. *Science* **2005**, *309*, 1864-1868
2. Wang, X.; Lan, J.; Ge, J.; Yu, J.; Shan, S. Crystal structure of SARS-CoV-2 spike receptor-binding domain bound with ACE2 receptor. *Protein Data Bank* **2020**.
3. Wrobel, A.G.; Benton, D.J.; Roustan, C.; Borg, A.; Hussain, S.; Martin, S.R.; Rosenthal, P.B.; Skehel, J.J.; Gamblin, S.J. Evolution of the SARS-CoV-2 spike protein in the human host. *Nature communications* **2022**, *13*, 1-7.

4. Wang, Y.; Liu, C.; Zhang, C.; Wang, Y.; Hong, Q.; Xu, S.; Li, Z.; Yang, Y.; Huang, Z.; Cong, Y. Structural basis for SARS-CoV-2 Delta variant recognition of ACE2 receptor and broadly neutralizing antibodies. *Nature Communications* **2022**, *13(1)*, 871.
5. Dejnirattisai, W.; Zhou, D.; Supasa, P.; Liu, C.; Mentzer, A.J.; Ginn, H.M.; Zhao, Y.; Duyvesteyn, H.M.; Tuekprakhon, A.; Nutalai, R. Antibody evasion by the P. 1 strain of SARS-CoV-2. *Cell* **2021**, *184*, 2939-2954. e2939.
6. Wang, Y.; Xu, C.; Wang, Y.; Hong, Q.; Zhang, C.; Li, Z.; Xu, S.; Zuo, Q.; Liu, C.; Huang, Z. Conformational dynamics of the Beta and Kappa SARS-CoV-2 spike proteins and their complexes with ACE2 receptor revealed by cryo-EM. *Nature Communications* **2021**, *12*, 1-13.
7. Han, P.; Li, L.; Liu, S.; Wang, Q.; Zhang, D.; Xu, Z.; Han, P.; Li, X.; Peng, Q.; Su, C. Receptor binding and complex structures of human ACE2 to spike RBD from Omicron and Delta SARS-CoV-2. *Cell* **2022**, *185*, 630-640. e610.
8. Maier, J.A.; Martinez, C.; Kasavajhala, K.; Wickstrom, L.; Hauser, K.E.; Simmerling, C. ff14SB: improving the accuracy of protein side chain and backbone parameters from ff99SB. *Journal of Chemical Theory & Computation Jctc* **2015**, *11*, 3696-3713.
9. Delano, W.L. The PyMol Molecular Graphics System. *Proteins Structure Function Bioinformatics* **2002**, *30*, 442-454.
10. Abraham, M.J.; Murtola, T.; Schulz, R.; Páll, S.; Smith, J.C.; Hess, B.; Lindahl, E. GROMACS: High performance molecular simulations through multi-level parallelism from laptops to supercomputers. *SoftwareX* **2015**, *1*, 19-25.
11. Jorgensen, W.L.; Chandrasekhar, J.; Madura, J.D.; Impey, R.W.; Klein, M.L. Comparison of simple potential functions for simulating liquid water. *The Journal of chemical physics* **1983**, *79*, 926-935.
12. Essmann, U.; Perera, L.; Berkowitz, M.L.; Darden, T.; Lee, H.; Pedersen, L.G. A smooth particle mesh Ewald method. *The Journal of chemical physics* **1995**, *103*, 8577-8593.
13. Bussi, G.; Donadio, D.; Parrinello, M. Canonical sampling through velocity rescaling. *The Journal of chemical physics* **2007**, *126*, 014101.
14. Valdés-Tresanco, M.S.; Valdés-Tresanco, M.E.; Valiente, P.A.; Moreno, E. gmx_MMPBSA: a new tool to perform end-state free energy calculations with GROMACS. *Journal of Chemical Theory Computational* **2021**, *17*, 6281-6291.

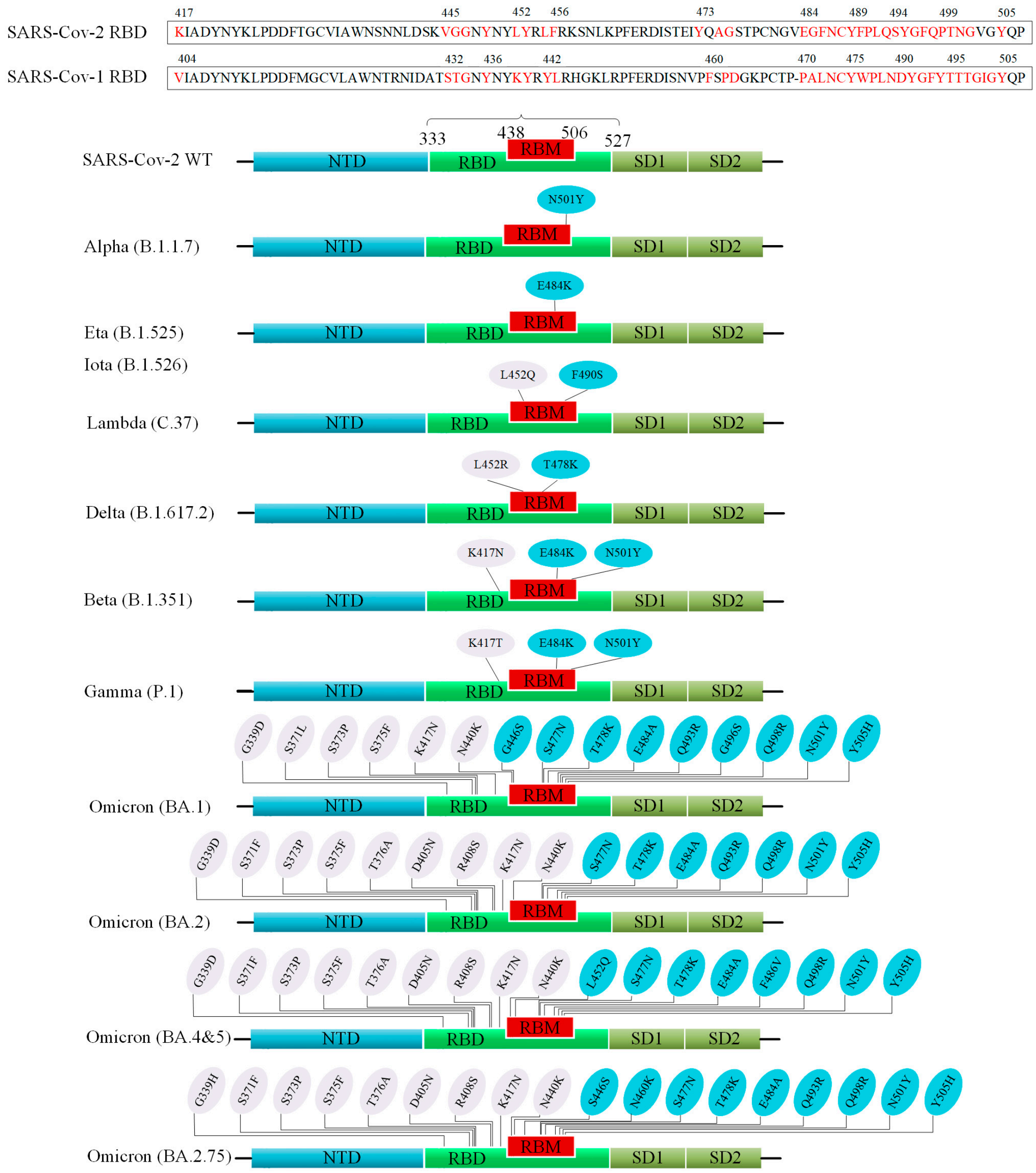

Figure S1 The mutations of SARS-CoV-1 and 11 different SARS-CoV-2 variants are of interest to scientists. The above is a comparison of the differences of amino acid sequences between SARS-CoV-1 and SARS-CoV-2. The below is the amino acid information of SARS-CoV-2 variants. The key mutations in RBM region are marked in light blue.

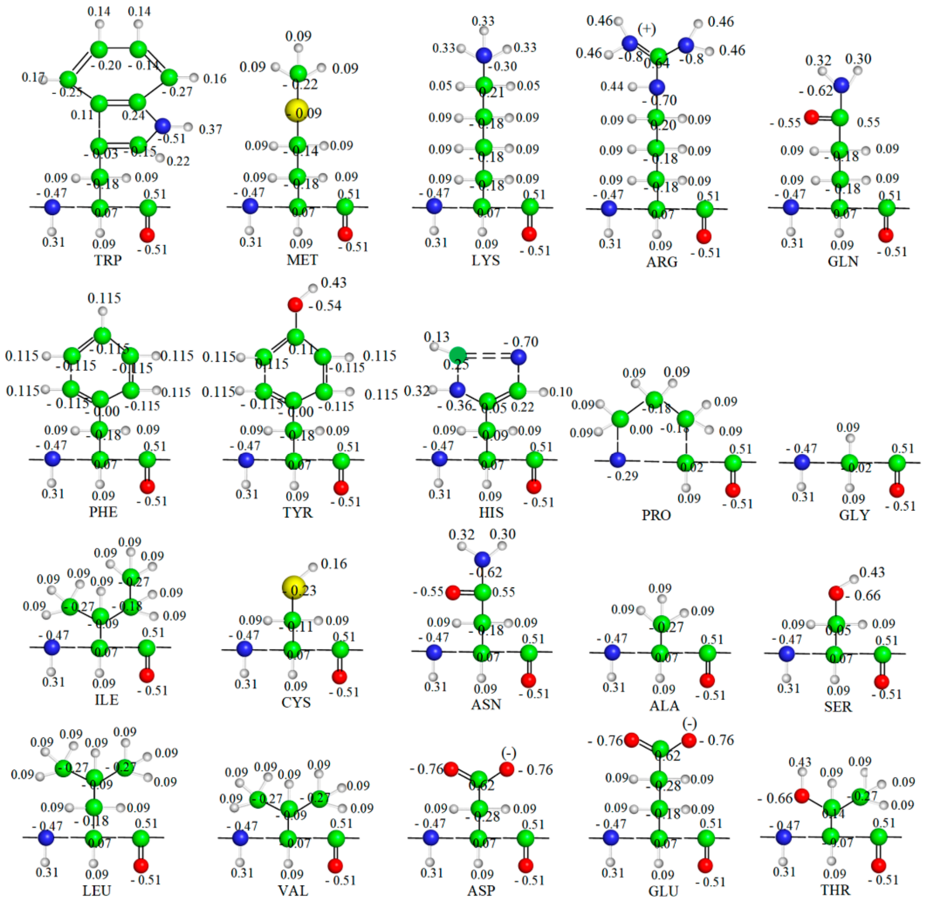

Figure S2 The partial charges based on the topological structures in charmm36 force field. Carbon, oxygen, nitrogen, sulphur, and hydrogen atoms are highlighted by green, red, blue, yellow, and white, respectively.

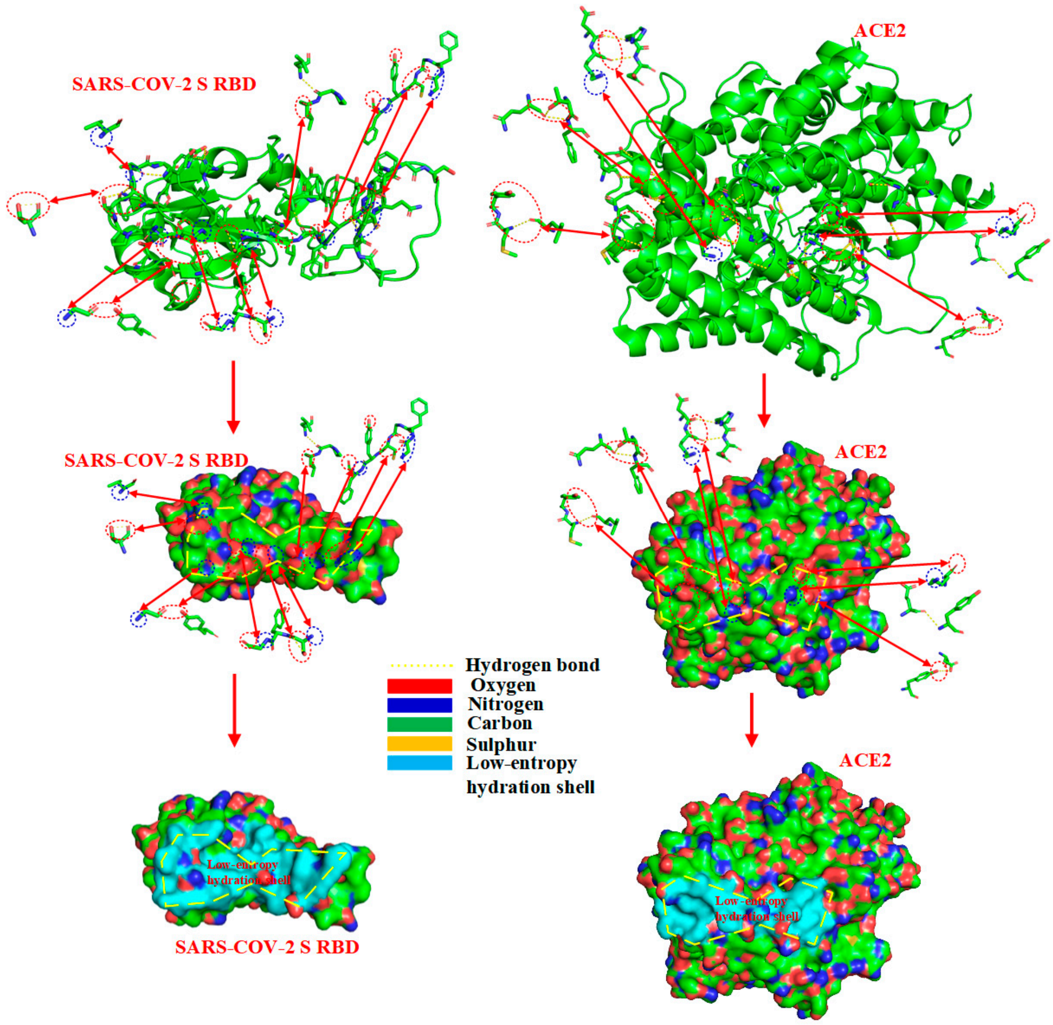

Figure S3 Low-entropy hydration of hydration shells on the binding sites of SARS-CoV-2 RBD and ACE2. The binding sites of the two proteins are highlighted by yellow dashed lines and the low-entropy hydration shell region is shown in cyan. The hydrophilic groups at the binding sites that do not express their hydrophilicity are indicated by red arrows. Carbon, oxygen, nitrogen, sulphur, and hydrogen atoms are highlighted by green, red, and blue, respectively.

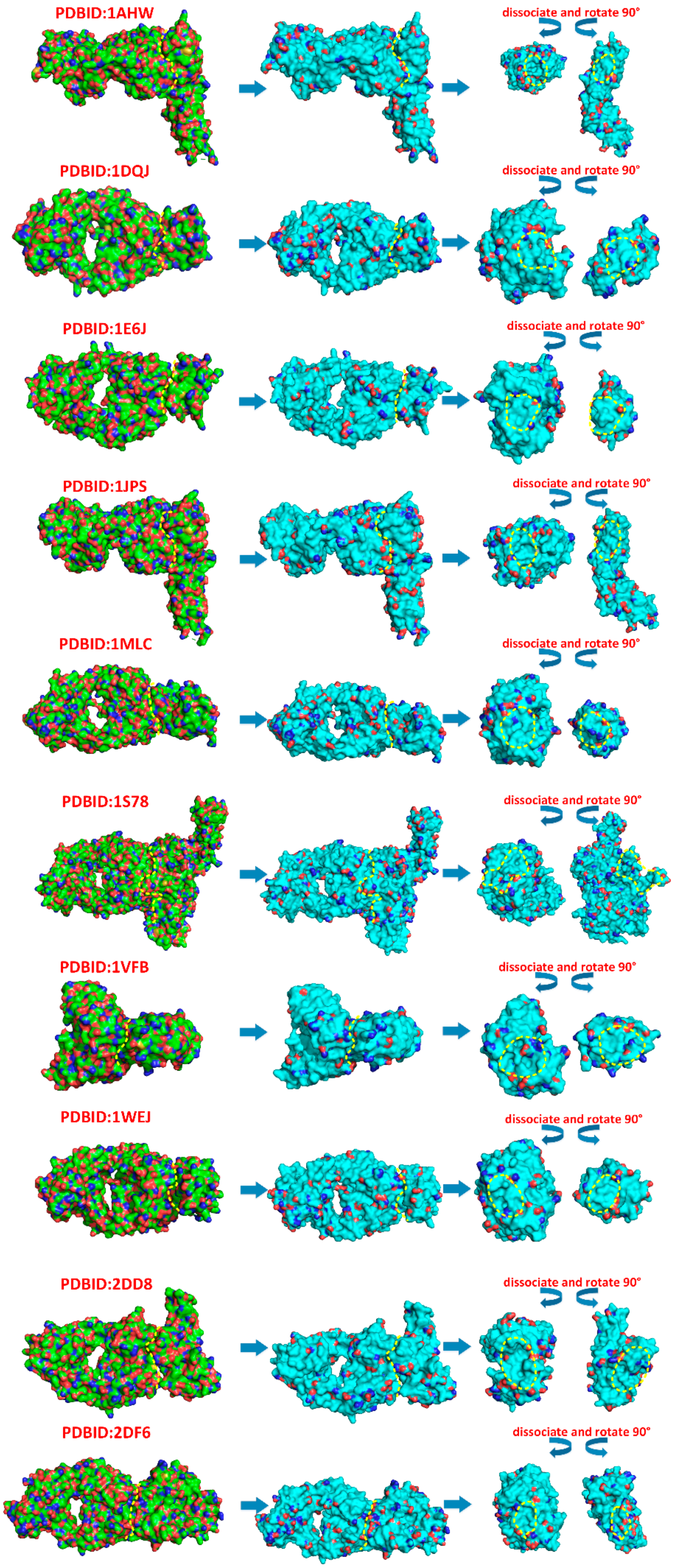

PDBID:1AHW
dissociate and rotate 90°
PDBID:1DQJ
dissociate and rotate 90°
PDBID:1E6J
dissociate and rotate 90°
PDBID:1JPS
dissociate and rotate 90°
PDBID:1MLC
dissociate and rotate 90°
PDBID:1S78
dissociate and rotate 90°
PDBID:1VFB
dissociate and rotate 90°
PDBID:1WEJ
dissociate and rotate 90°
PDBID:2DD8
dissociate and rotate 90°
PDBID:2DF6
dissociate and rotate 90°

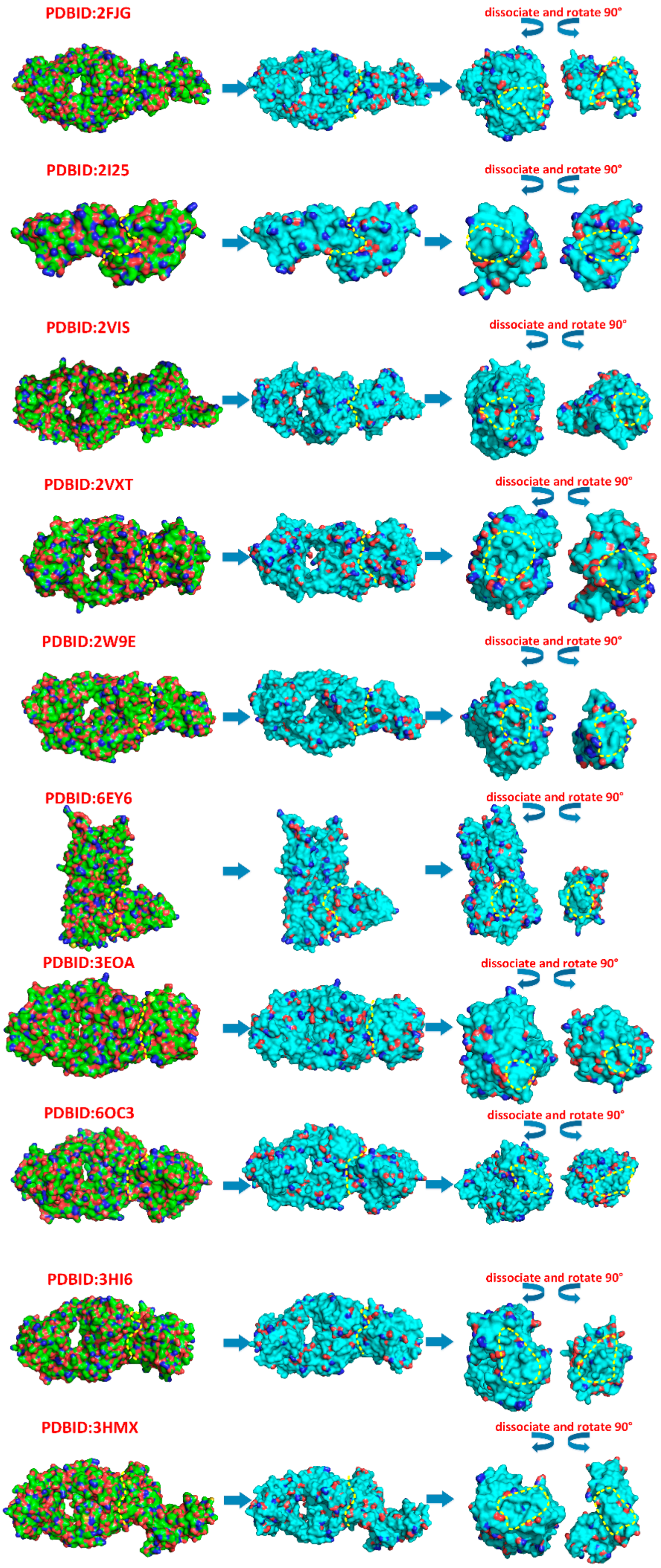
PDBID:2FJG
dissociate and rotate 90°
PDBID:2I25
dissociate and rotate 90°
PDBID:2VIS
dissociate and rotate 90°
PDBID:2VXT
dissociate and rotate 90°
PDBID:2W9E
dissociate and rotate 90°
PDBID:6EY6
dissociate and rotate 90°
PDBID:3EOA
dissociate and rotate 90°
PDBID:6OC3
dissociate and rotate 90°
PDBID:3HI6
dissociate and rotate 90°
PDBID:3HMX
dissociate and rotate 90°

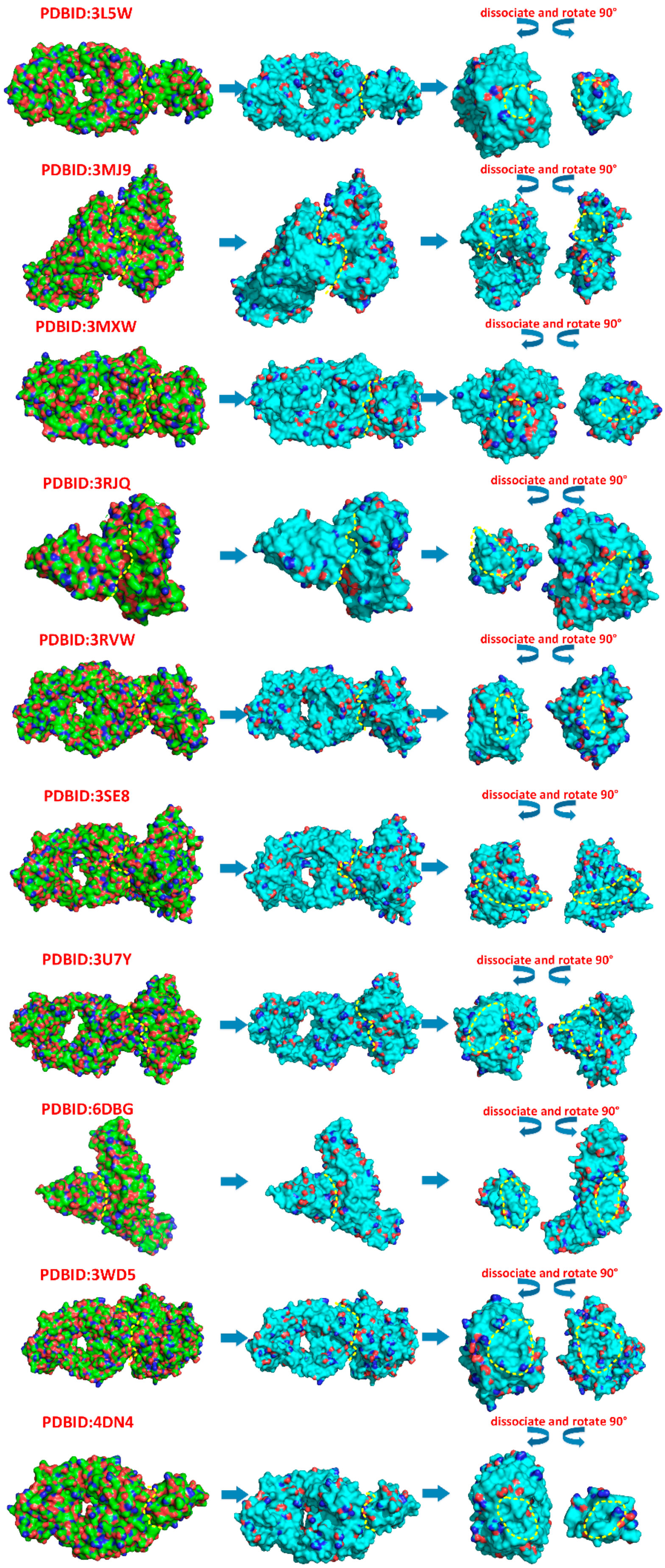
PDBID:3L5W
dissociate and rotate 90°
PDBID:3MJ9
dissociate and rotate 90°
PDBID:3MXW
dissociate and rotate 90°
PDBID:3RJQ
dissociate and rotate 90°
PDBID:3RVW
dissociate and rotate 90°
PDBID:3SE8
dissociate and rotate 90°
PDBID:3U7Y
dissociate and rotate 90°
PDBID:6DBG
dissociate and rotate 90°
PDBID:3WD5
dissociate and rotate 90°
PDBID:4DN4
dissociate and rotate 90°

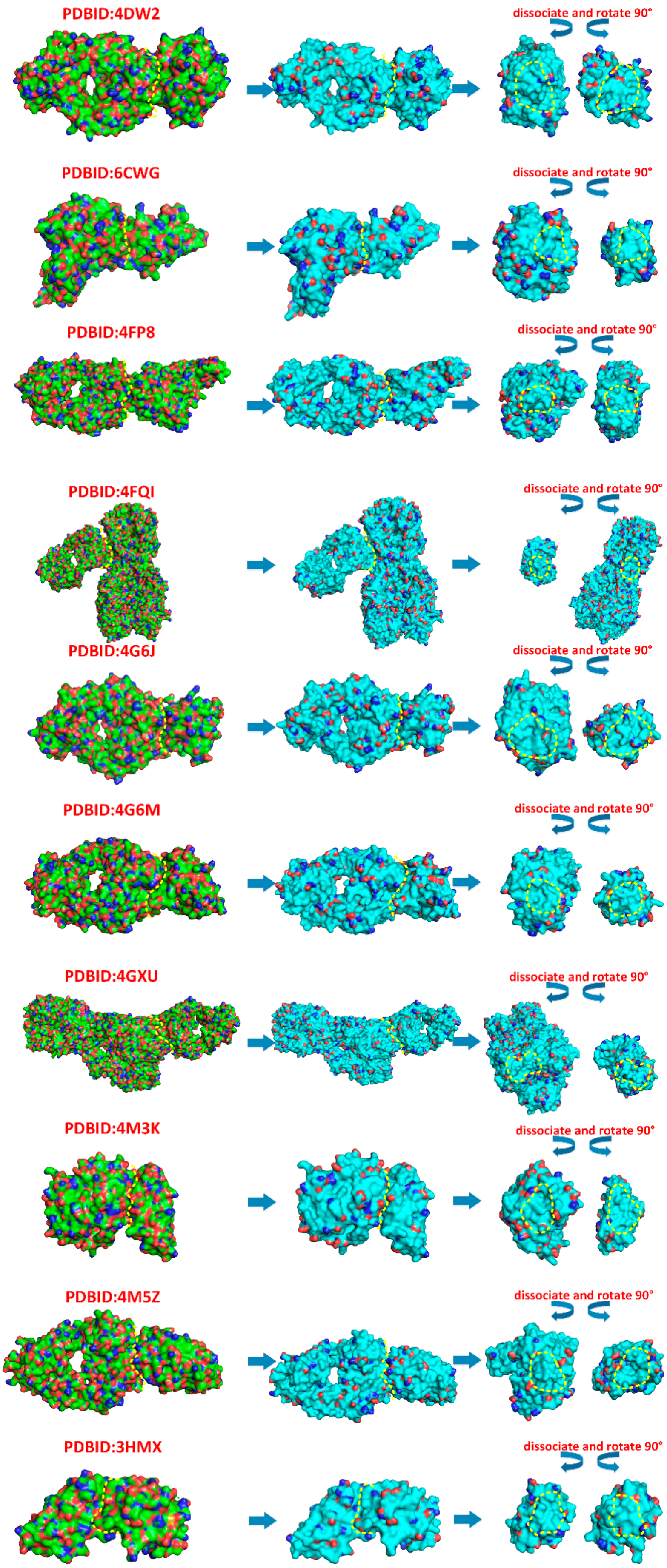

PDBID:4DW2
dissociate and rotate 90°
PDBID:6CWG
dissociate and rotate 90°
PDBID:4FP8
dissociate and rotate 90°
PDBID:4FQI
dissociate and rotate 90°
PDBID:4G6J
dissociate and rotate 90°
PDBID:4G6M
dissociate and rotate 90°
PDBID:4GXU
dissociate and rotate 90°
PDBID:4M3K
dissociate and rotate 90°
PDBID:4M5Z
dissociate and rotate 90°
PDBID:3HMX
dissociate and rotate 90°

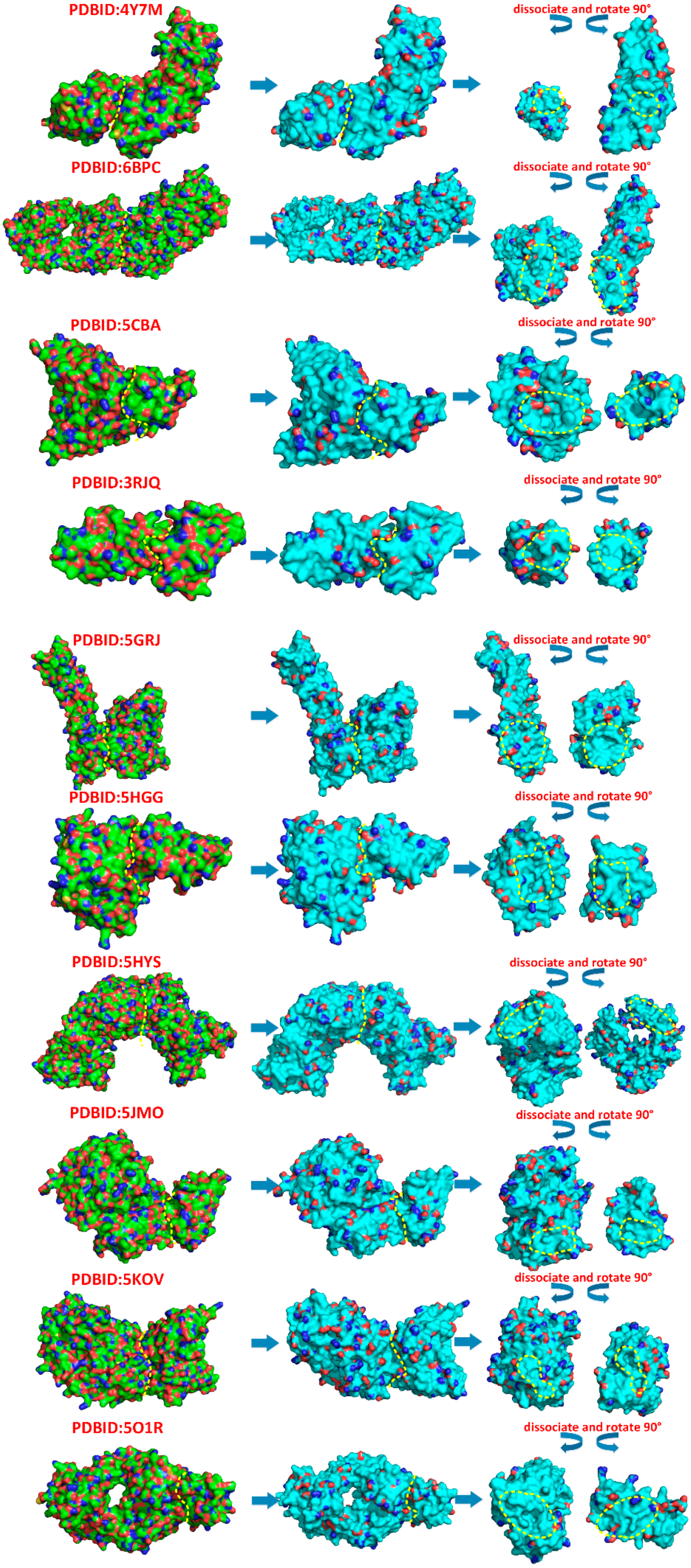
PDBID:4Y7M
dissociate and rotate 90°
PDBID:6BPC
dissociate and rotate 90°
PDBID:5CBA
dissociate and rotate 90°
PDBID:3RJQ
dissociate and rotate 90°
PDBID:5GRJ
dissociate and rotate 90°
PDBID:5HGG
dissociate and rotate 90°
PDBID:5HYS
dissociate and rotate 90°
PDBID:5JMO
dissociate and rotate 90°
PDBID:5KOV
dissociate and rotate 90°
PDBID:5O1R
dissociate and rotate 90°

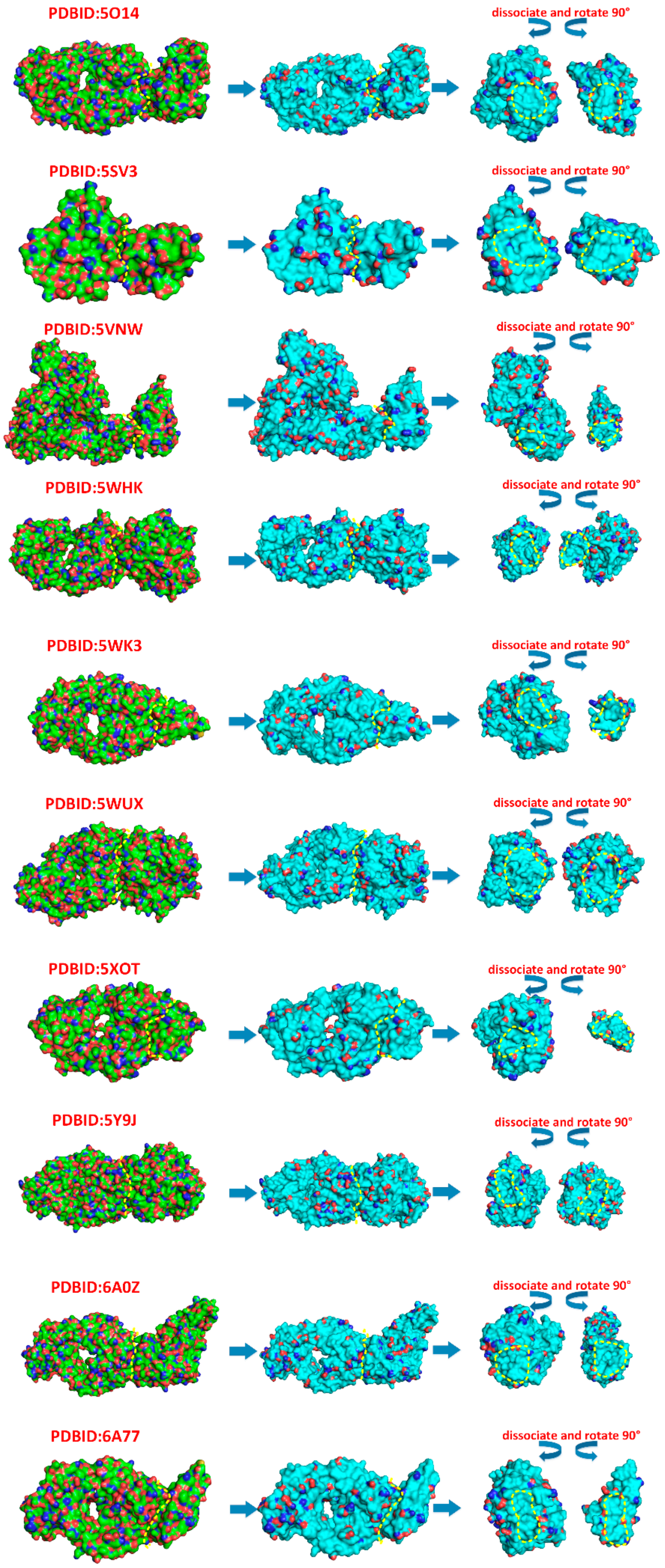
PDBID:5O14
dissociate and rotate 90°
PDBID:5SV3
dissociate and rotate 90°
PDBID:5VNW
dissociate and rotate 90°
PDBID:5WHK
dissociate and rotate 90°
PDBID:5WK3
dissociate and rotate 90°
PDBID:5WUX
dissociate and rotate 90°
PDBID:5XOT
dissociate and rotate 90°
PDBID:5Y9J
dissociate and rotate 90°
PDBID:6A0Z
dissociate and rotate 90°
PDBID:6A77
dissociate and rotate 90°

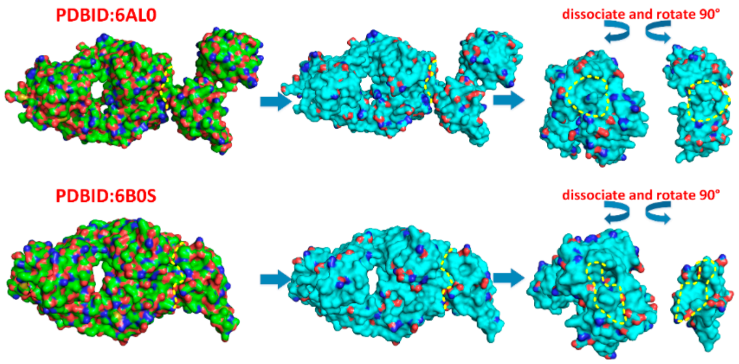

Figure S4 Low–entropy hydration shells at binding sites guide protein binding using 62 antibody–antigen test cases for Docking Benchmark 5.5. The identification of binding sites of protein subunits for antibody–antigen test cases by detecting relatively large low–entropy regions of hydration shells of individual protein subunits. The low–entropy hydration shell regions are shown in cyan, and the curves and irregular circles marked with yellow dashed lines represent the binding surface and binding sites, respectively.

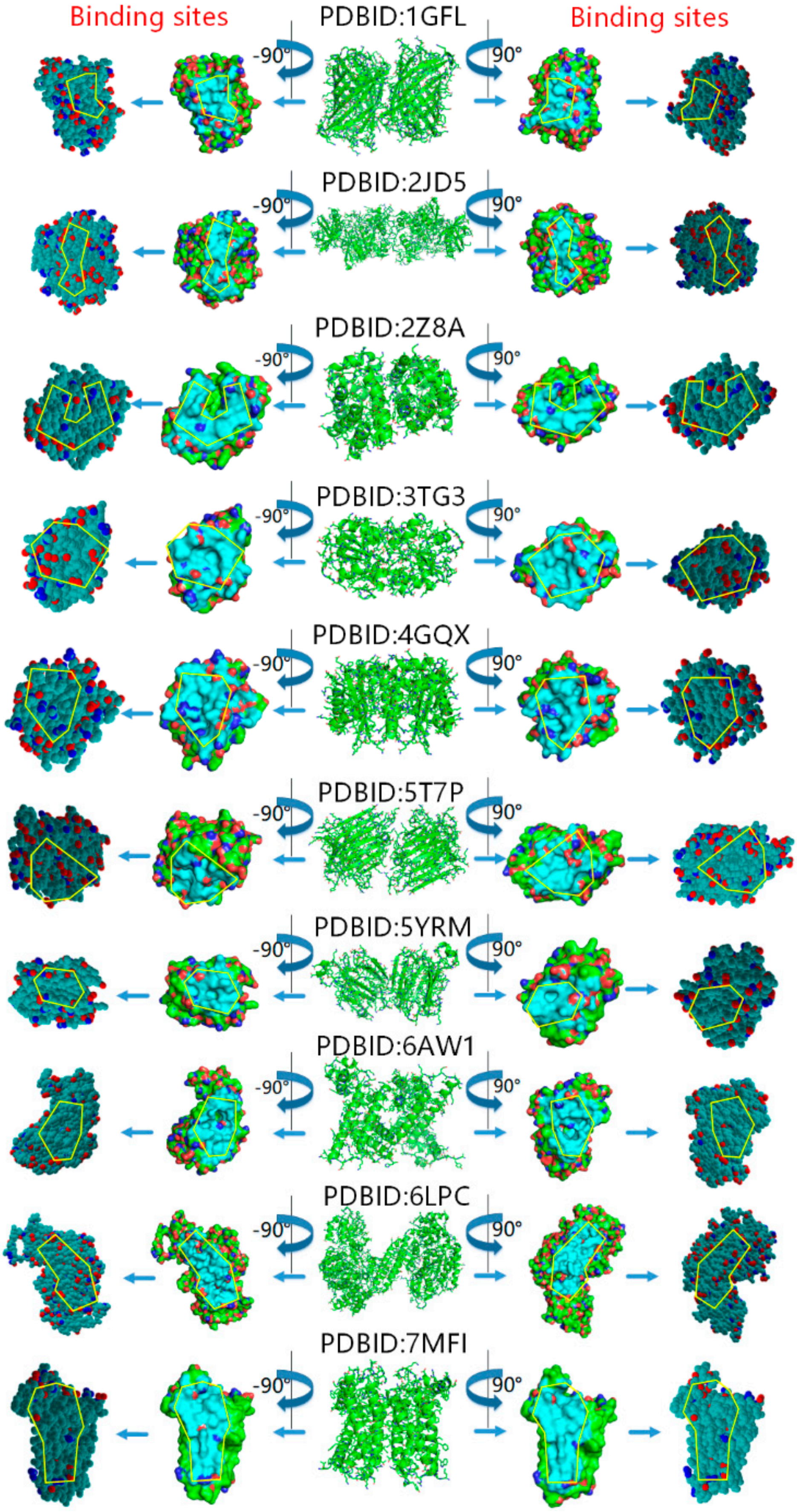

Binding sites
PDBID:1GFL
Binding sites
-90°
90°
PDBID:2JD5
PDBID:2Z8A
PDBID:3TG3
PDBID:4GQX
PDBID:5T7P
PDBID:5YRM
PDBID:6AW1
PDBID:6LPC
PDBID:7MFI

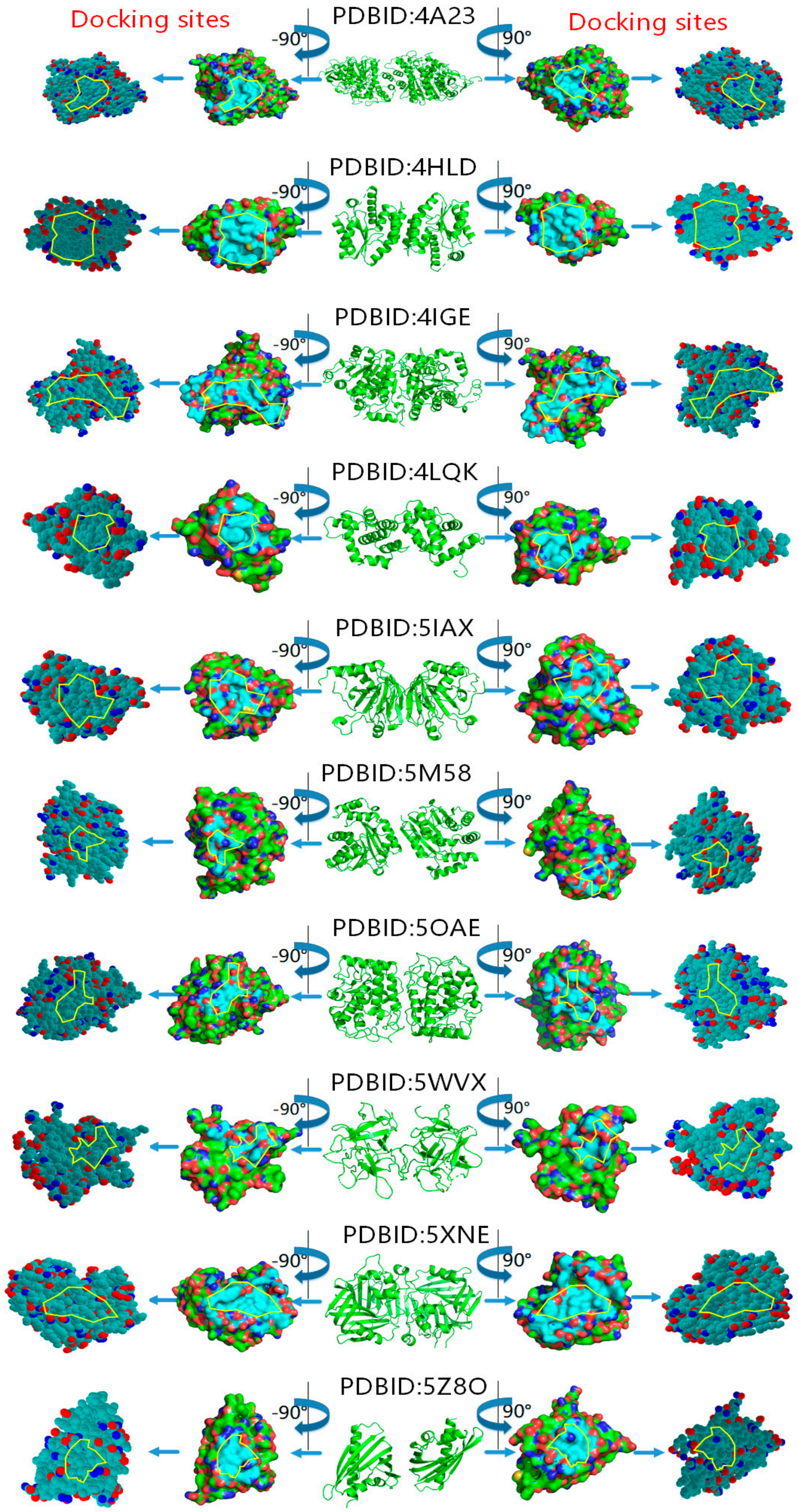

Docking sites
Docking sites
PDBID:4A23
PDBID:4HLD
PDBID:4IGE
PDBID:4LQK
PDBID:5IAX
PDBID:5M58
PDBID:5OAE
PDBID:5WVX
PDBID:5XNE
PDBID:5Z8O
-90°
90°

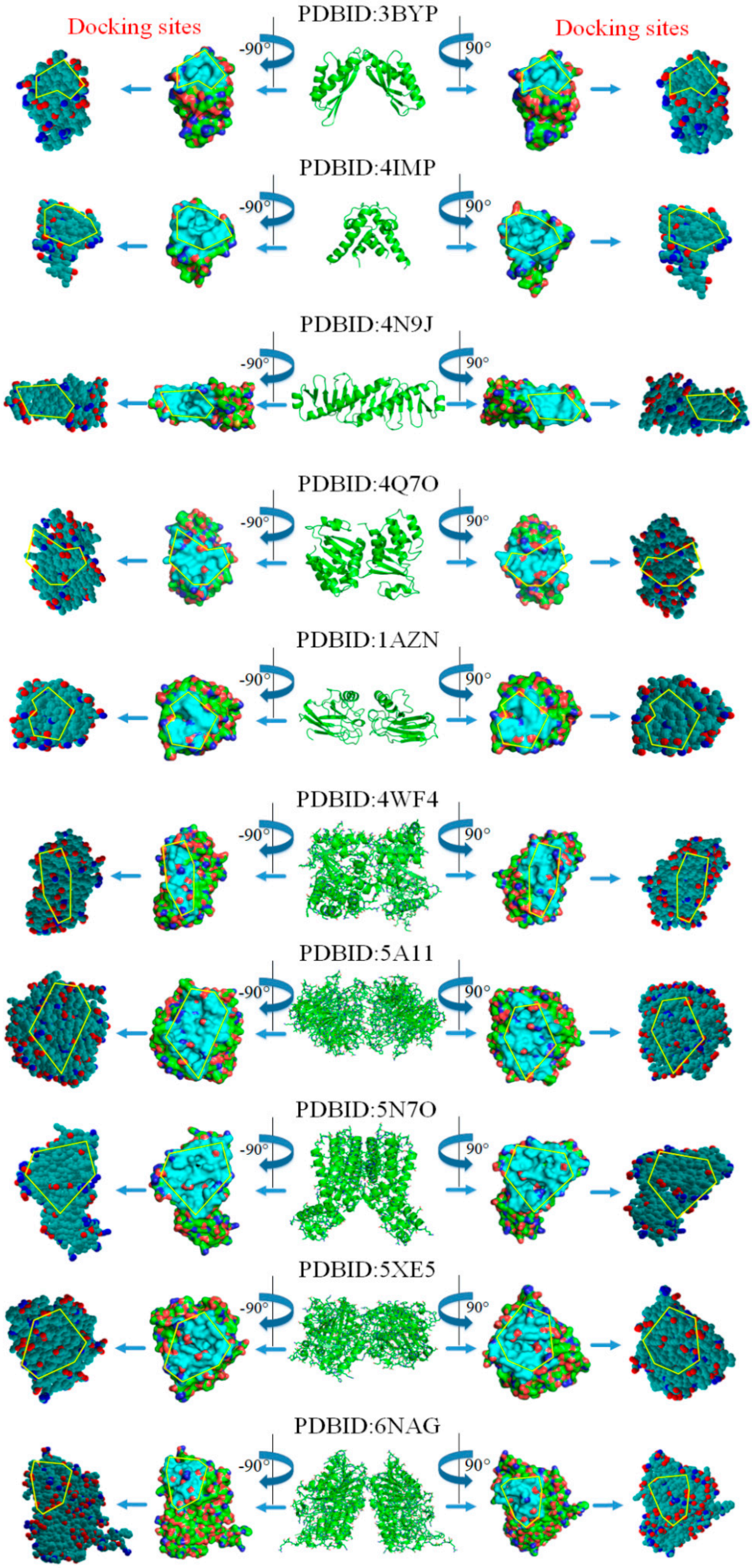
PDBID:3BYP
Docking sites
Docking sites
-90°
90°
PDBID:4IMP
-90°
90°
PDBID:4N9J
-90°
90°
PDBID:4Q7O
-90°
90°
PDBID:1AZN
-90°
90°
PDBID:4WF4
-90°
90°
PDBID:5A11
-90°
90°
PDBID:5N7O
-90°
90°
PDBID:5XE5
-90°
90°
PDBID:6NAG
-90°
90°

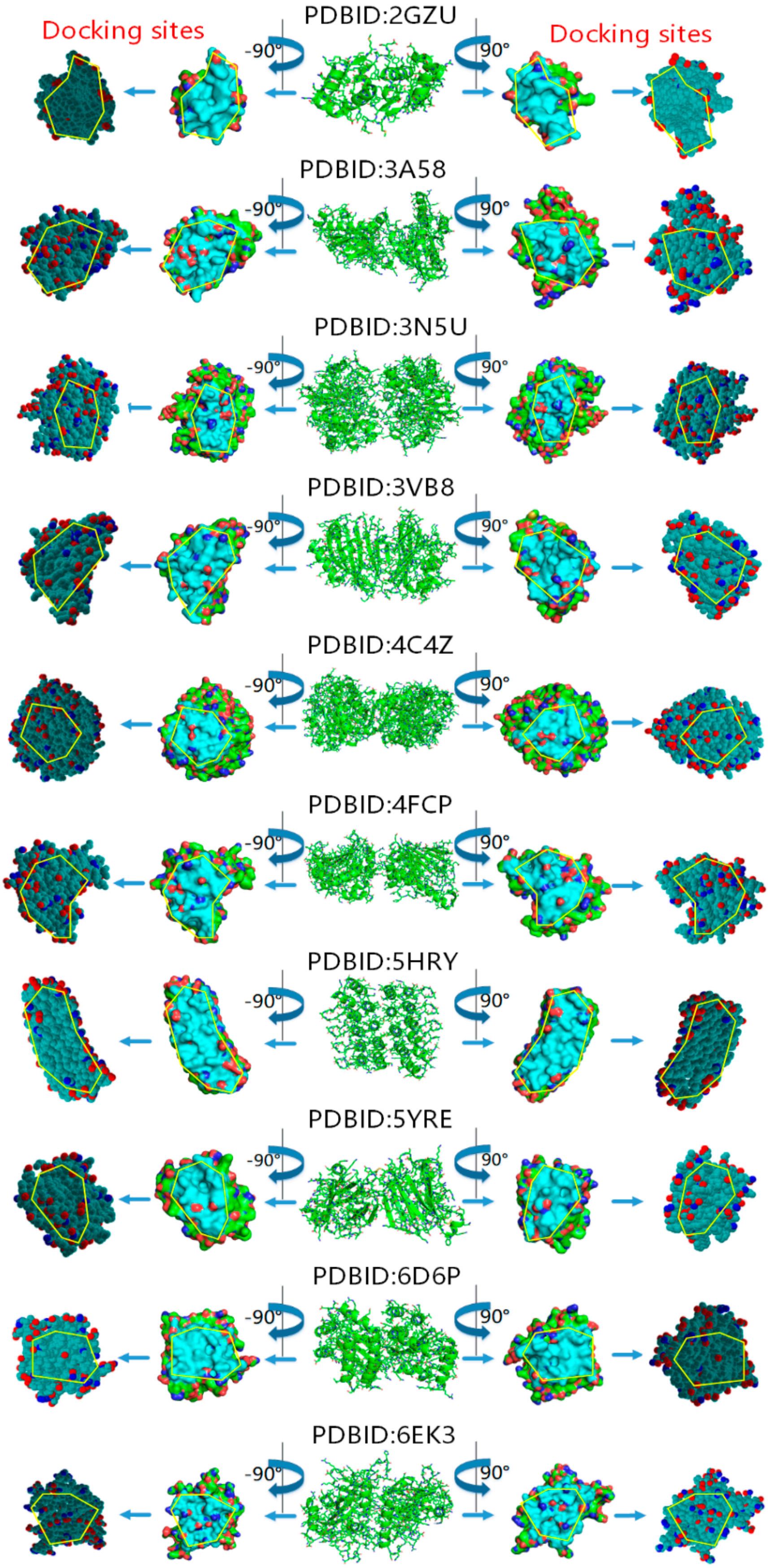

Docking sites
Docking sites
PDBID:2GZU
PDBID:3A58
PDBID:3N5U
PDBID:3VB8
PDBID:4C4Z
PDBID:4FCP
PDBID:5HRY
PDBID:5YRE
PDBID:6D6P
PDBID:6EK3
-90°
90°

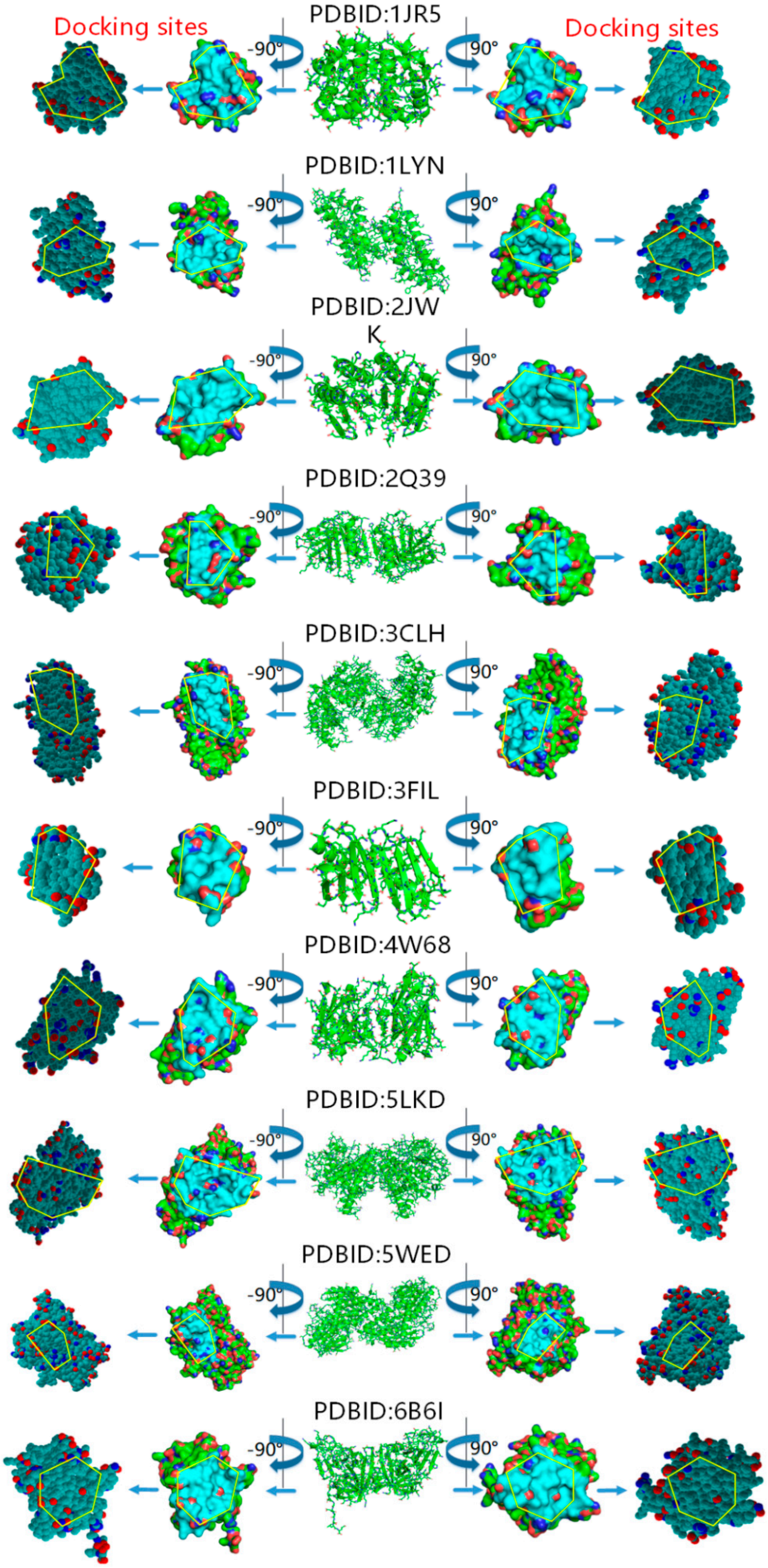

PDBID:1JR5
Docking sites
Docking sites
-90°
90°
PDBID:1LYN
-90°
90°
PDBID:2JW
K
-90°
90°
PDBID:2Q39
-90°
90°
PDBID:3CLH
-90°
90°
PDBID:3FIL
-90°
90°
PDBID:4W68
-90°
90°
PDBID:5LKD
-90°
90°
PDBID:5WED
-90°
90°
PDBID:6B6I
-90°
90°

Figure S5 The prediction of binding sites of protein subunits for 50 protein quaternary structures through identifying the low-entropy regions of hydration shells of individual protein subunits. The low–entropy hydration shell regions are shown in cyan, and irregular circles marked with yellow lines represent the binding surface.

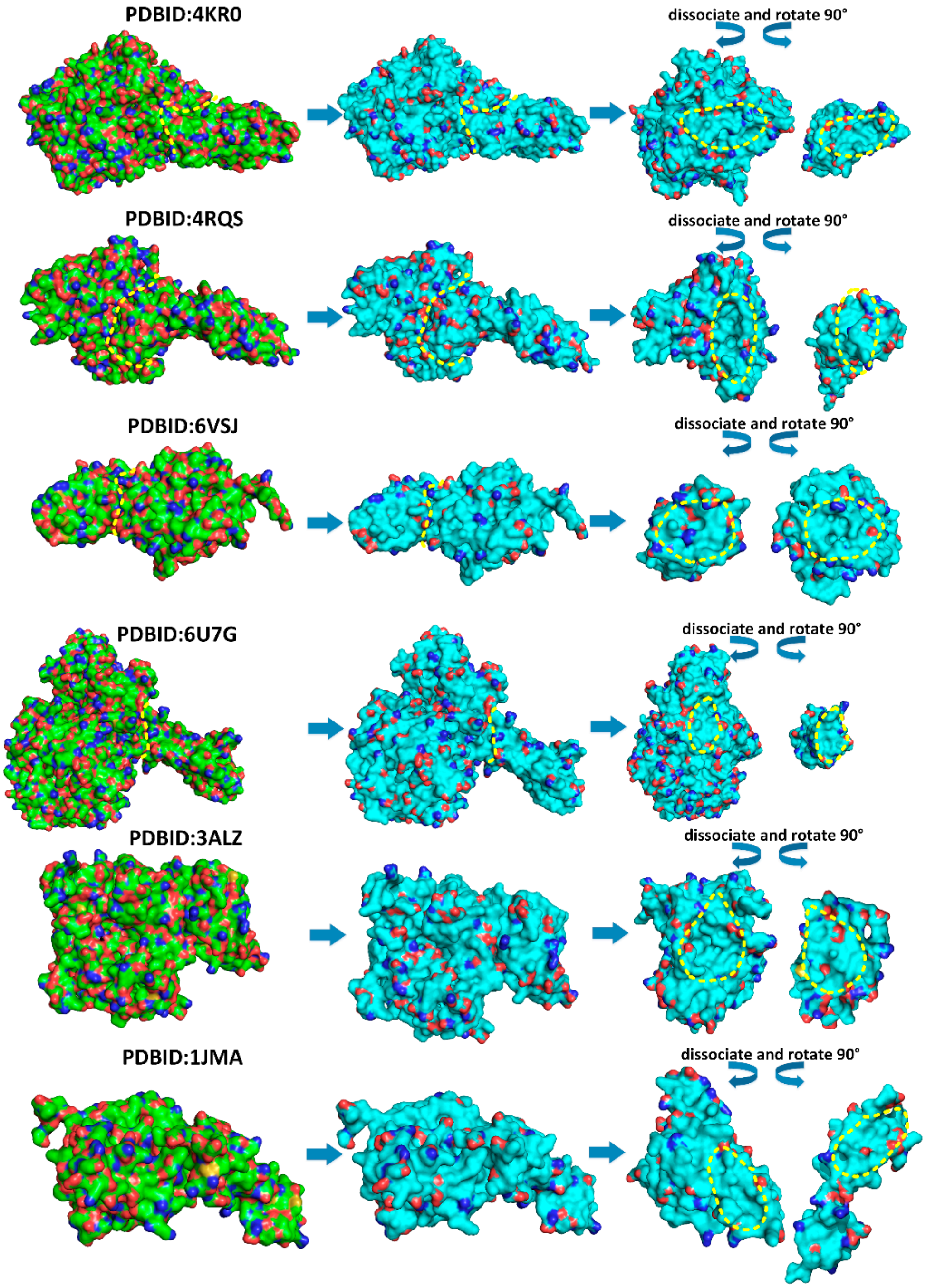

Figure S6. The low-entropy region at the binding sites of HIV (PDBID: 4RQS), MERS (PDBID: 4KR0), mouse coronavirus (PDBID: 6VSJ), measles virus hemagglutinin (PDBID: 3ALZ), human coronavirus HCoV-229E (PDBID: 6U7G), and herpes (PDBID: 1JMA). The low-entropy regions of the hydration shells are shown in cyan.

Table S1 The statistical results of RBD-antibodies binding sites at 200 experimentally determined structures.

PDBID	Antibodies binding sites	SARS-CoV-2	Antibodies numbering
7TBF	1 left, 1 right	WT	B1-182.1 and A19-61.1
7KZB	1 left, 2	WT	CR3014-C8 and CR3022-B6
8DW3	1left, 2, 5	WT	CR3022, S309 and SR16m
6WPT	5	WT	S309
7M7W	2	WT	S2X259 and S2H97
7R6W	2, 5	WT	S309 and S2X35
7R6X	1left, 2, 5	WT	S304 and S309 and S2E12
7R7N	1 left and 3 upper	WT	S2D106 FAB
7M42	1, 2	WT	REGN10989 and REGN10985
7E3O	1 middle and 3 upper	WT	nCoV617
7M6I	1	WT	BG1-24
7M6E	4 and 5	WT	BG10-19
7M6D	1, 2	WT	CR3022 and BG4-25
7B3O	1	WT	STE90-C11
7DPM	2	WT	MW06
7X7O	1 left	WT	UT28K
7WOG	3 and 5	Omicron	553-49
7E23	1 middle and 3 upper	WT	CA521
7KS9	1	WT	910-30
7E5O	1	WT	NT-193
7VYR	1 right	WT	D27
7WO7	2	WT	mAb15
7WOC	3 upper	WT	mAb60
7AKJ	4	WT	47D11
7A5R	1	WT	CR3022
7XH8	1 left	Omicron	ZCB11
7KFX	1	WT	C1A-C2
7KFY	1	WT	C1A-F10
7KFW	1	WT	C1A-B3
7KFV	1	WT	C1A-B12
7JMW	2	WT	COVA1-16
7CAH	2	WT	H014
7JX3	1, 2, 5	WT	S309, S2H14 and S304
8HED	1 right	WT	9H1
7KLH	1	WT	15033-7
7K45	1 left	WT	S2E12
7K43	1 middle	WT	S2M11
8BSE	1	WT	1D1
7N5H	2	WT	2-36
6XDG	1 left, 1 right	WT	REGN10933 and REGN10987
7SN2	2	WT	C1C-A3

7CDJ	1	WT	P2C-1A3
7CDI	1	WT	P2C-1F11
8DZH	1 right	BA.1.1.529	MB.02
7N4J	1	WT	WRAIR-2173
7N4I	3	WT	WRAIR-2057
7N4L	1 left	WT	WRAIR-2125
7N4M	2	WT	WRAIR-2151
7L7D	1 left	WT	AZD8895
7L7E	1 left, 1 right	WT	AZD8895 and AZD1061
7DJZ	1 middle	WT	MW01
7DK0	2	WT	MW05
6W41	2	WT	CR3022
7XSW	5	WT	S309
7XNF	1	WT	P2C-1F11
7CYP	1 right	WT	HB27
7CHP	1	WT	P5A-3C8
7CHS	1	WT	P22A-1D1
7CHO	1	WT	P5A-1D2
7FCQ	2	WT	P14-44
7YAD	5	Omicron	S309
6XE1	1	WT	CV30
7JN5	2	WT	CR3022
7R8L	1, 2	WT	C099 and CR3022
7N3I	1	WT	C098
7R8M	1 right	WT	C032
7R8N	1	WT	C051
7R8O	1 middle	WT	C548
7X7T	2, 3	WT	X01, X10 and X17
7X7U	2, 3	Delta	X01, X10 and X17
7KGK	1	WT	Sb16
7KGJ	1	WT	Sb45
7KLW	1, 2	WT	Sb45 and Sb68
7MFU	1, 2	WT	Sb14 and Sb68
7TAS	1	WT	S2K146
7E8M	1	WT	P2C-1F11
7DX4	3 and 5	WT	FC08
8GB7	1 right	WT	20A7
7EAN	3	WT	6D6
7K8M	1	WT	C102
8GB8	1 right	BA.2	20A7
7QTK	5	B.1.1.529	P2G3
7QTI	5	B.1.1.529	P2G3
7KMI	1	WT	LY-CoV481
8DXU	1 left, 3 and 5	WT	10G4 and GAR03

8DXT	1	WT	GAR12
6XKP	5	WT	CV07-270
6XKQ	1	WT	CV07-250
8GS9	1	BA.2	VacBB-551
8F0H	1, 3	WT	1H2 and 2A10
8F0G	1 left	BA.1	1C3
8A96	1	WT	Fab47
8HED	1 right	WT	9H1
7XS8	1	WT	P5S-1H1
7XIL	1	Beta	B38
7XIK	1	Omicron	B38
8I5I	1	Delta	NCV2SG53
7WN2	1	WT	NCV2SG53
7YOW	1	Omicron	NCV2SG48
7WNB	1	WT	NCV2SG48
8I5H	1	Delta	NCV2SG48
8C1V	2	WT	Sb92
7UOW	1	WT	034_32
8GZZ	5	BA.1	1H1
8GJN	1 left	WT	17B10
8BBN	2, 5	Delta	EY6A and BA.2-10
8BBO	5	Delta	BA.2-36
8BCZ	3 and 5, 2, 1, 5	Delta	BA.2-36, BA.2-23, EY6A, COVOX-45
8C3V	2, 5	Delta	BA.2-13 and C1
7X96	4 upper and 5	WT	Ab847
7X95	1	WT	Ab709
7X94	1	WT	Ab712
7X93	1 left	WT	Ab765
7XCZ	1, 5 upper	Delta	BA7054 and BA7125
7XDA	1, 5 upper	Delta	BA7208 and BA7125
7XDB	5	Omicron	BA7208
7U8E	2	WT	Ab246
7X66	1	Omicron	BD-236
7X63	1	Beta	BD-236
7UL0	1 left	WT	EH8
7UL1	1	WT	EH3
7WP8	3 2 5	WT	83H7
7X2H	4 upper and 1 right	WT	6-2C
7WSC	2	Omicron	BD55-3500
7U0Q	1	WT	002-02
7U0X	2	WT	002-13
7WP6	1 left, 3, 5	WT	36H6, 83H7, 85F7
8C8P	1 left	WT	10D12
7Y7K	1 left	WT	1F

7YCK	2	WT	FP-12A
7YCL	2	WT	IS-9A
7YCN	2	WT	IY-2A
8HC8	3	BA.1	YB13-292
8HC6	1	BA.1	YB9-258
8HC5	1, 3	WT	YB9-258 and R1-32
8BE1	1 left	WT	mRBD2
6YLA	2	WT	CR3022
7UZC	2	WT	M8a-34
7UZA	1 left	WT	HSW-1
7UZ9	2	WT	M8a-34
7UZ8	2	BA.1	M8a-31
7UZ7	2	WT	M8a-31
7UZ6	5	WT	M8a-28
7UZ5	2	WT	M8a-6
7UZ4	2	WT	M8a-3
7WCU	1	B.1.617.2	SWC11
7WCK	1 left	B.1.617.2	SWA9
7X2K	1 left, 2	WT	1F11 and Nb70
8GZ5	1	WT	P17
8DWA	5	WT	P1D9
8DW9	5	WT	D29
8DXS	1 right	WT	P2B4
7YKJ	1 left	Omicron	P3E6
7X8W	1	WT	Ab354
7X8Y	1 left	WT	Ab159
7X8Z	1 left	WT	Ab188
7X90	1 left and 3 upper	WT	Ab326
7X91	1	WT	Ab496
7X92	1	WT	Ab445
7WVL	1 left	WT	P4A2
7WOP	1 left and 2	Omicron	FD01
7S83	3, 2	WT	ShAb01 and ShAb02
8GB6	5	WT	21B6
7U9O	1 left and 3 upper	WT	NE12
7U9P	5	WT	NA8
7XST	1, 5 and 5 upper	Omicron	F61 and D2
7SIX	2	WT	N3-1
8GB5	2	WT	25F9
8ERQ	1 right and 5 upper	BA.1	S2X324
8D8R	1 left	WT	DMAb 2196
8D8Q	1 left, 5	WT	DMAbs 2130 and 2196
7YR0	5	BA.2.75	S309
8DNN	1 left	WT	80 FAB

8GX9	1 left, 5	WT	P2C-1F11 and P2B-1G5
7ZCF	1	WT	scFv76
7XRP	5	WT	C5G2
7BZ5	1	Delta	B38
7Y0C	5	Omicron	BD55-1403
7Y0V	5	BA.1	5549
7Y0W	1 right, 5	BA.1	BD55-5514 and BD55-5840
7WRJ	1 right and 3	B.1.1.529	BD55-4637
7WPH	5	WT	Fab06
7XXL	1 left	WT	Fab14
8CWK	1 left, 3	WT	4G1-C2 and 10G4
8CWJ	2, 3	WT	4C12-B12 and CR3022
7XCK	5	Omicron	S309
7WBZ	1	WT	2303
8DAD	1 left	WT	AZ090
8DI5	1	Beta	VH F6
7YDI	3 and 5	WT	R1-32
7RU4	4	WT	CC6.33
7RU8	1 left	WT	CC6.30
8CWI	1 left	WT	10G4
7WM0	5	Omicron	35B5
7UPX	5	WT	SP1-77
7UPL	5	Omicron	002-S21F2
7XSC	1	WT	P5S-2B10
7XSA	5	WT	3C11
7XSB	2	WT	P5S-3B11
7FJC	5	Beta	P36-5D2
7C01	1	WT	CB6
